# Kinetic Basis of the Bifunctionality of SsoII DNA Methyltransferase

**DOI:** 10.3390/molecules23051192

**Published:** 2018-05-16

**Authors:** Nadezhda A. Timofeyeva, Alexandra Yu. Ryazanova, Maxim V. Norkin, Tatiana S. Oretskaya, Olga S. Fedorova, Elena A. Kubareva

**Affiliations:** 1 Siberian Branch of the Russian Academy of Sciences, Institute of Chemical Biology and Fundamental Medicine, Lavrentyev Ave., 8, 630090 Novosibirsk, Russia; na_timof@niboch.nsc.ru (N.A.T.); fedorova@niboch.nsc.ru (O.S.F.); 2 Belozersky Institute of Physico-Chemical Biology and Chemistry Department, Lomonosov Moscow State University, Leninskye Gory, 1, 119991 Moscow, Russia; venel.ale@gmail.com (A.Yu.R.); maxim.norkin@epfl.ch (M.V.N.); oretskaya@belozersky.msu.ru (T.S.O.)

**Keywords:** restriction–modification system, DNA methyltransferase, enzyme kinetics, pre-steady-state kinetics, stopped-flow assay, transcription factor

## Abstract

Type II restriction–modification (RM) systems are the most widespread bacterial antiviral defence mechanisms. DNA methyltransferase SsoII (M.SsoII) from a Type II RM system SsoII regulates transcription in its own RM system in addition to the methylation function. DNA with a so-called regulatory site inhibits the M.SsoII methylation activity. Using circular permutation assay, we show that M.SsoII monomer induces DNA bending of 31° at the methylation site and 46° at the regulatory site. In the M.SsoII dimer bound to the regulatory site, both protein subunits make equal contributions to the DNA bending, and both angles are in the same plane. Fluorescence of TAMRA, 2-aminopurine, and Trp was used to monitor conformational dynamics of DNA and M.SsoII under pre-steady-state conditions by stopped-flow technique. Kinetic data indicate that M.SsoII prefers the regulatory site to the methylation site at the step of initial protein–DNA complex formation. Nevertheless, in the presence of *S*-adenosyl-l-methionine, the induced fit is accelerated in the M.SsoII complex with the methylation site, ensuring efficient formation of the catalytically competent complex. The presence of *S*-adenosyl-l-methionine and large amount of the methylation sites promote efficient DNA methylation by M.SsoII despite the inhibitory effect of the regulatory site.

## 1. Introduction

Viruses and virus-like selfish elements are ubiquitous on Earth [1,2]. All bacteria and archaea exist in a permanent arms race with viral parasites and therefore have developed various antiviral defence mechanisms. They include restriction–modification (RM) systems, DNA phosphorothioation, CRISPR/Cas, toxin–antitoxin, abortive infection (phage exclusion) as well as the recently discovered argonaute-based interference and BREX systems [3,4,5,6,7,8,9,10,11,12]. The genes encoding components of the defence systems are typically clustered in defence islands [13,14]. These genes efficiently spread among different organisms through horizontal gene transfer (by natural transformation or by means of mobile genetic elements) [15,16,17,18]. One organism can carry multiple defence systems of one or more classes. In some cases, these systems can operate synergistically and increase the overall cell resistance to phage infection [19]. The proportion of the genome occupied by the defence systems varies broadly among different bacteria and archaea, from ~0 to ~10% [14].

Among different defence systems, RM systems are the most well studied, and amidst the RM systems, Type II systems are the most actively explored because they are the most abundant in Nature and because they contain restriction enzymes necessary for genetic engineering [18]. A typical Type II RM system consists of a modification enzyme (DNA methyltransferase, MTase) and a restriction enzyme (restriction endonuclease, REase) that recognise the same sequence in DNA. MTase methylates this site while REase cleaves DNA if the site remains unmodified. Thus, RM systems protect their host cell from phage infections and can be regarded as prokaryotic innate immunity. The methyl group donor in methylation reactions is *S*-adenosyl-l-methionine (AdoMet), which is converted into *S*-adenosyl-l-homocysteine (AdoHcy) [3].

RM system SsoII is encoded in a ColE1-type plasmid P4 found in *Shigella sonnei* [20]. It consists of two divergent genes (*ssoIIM* encoding the MTase [21] and *ssoIIR* encoding the REase [R.SsoII]) separated by an intergenic region of 109 bp. The regions with 99% identity to the SsoII RM system are present in several other plasmids, namely, HSD from *Enterobacter cloacae* 18 k [22], pKPN2 from *Klebsiella pneumoniae* 2 k [23], pSTd4 from *Salmonella typhi* D4 [24], pFM366 from *Salmonella enteritidis* P1 [25] and pCE10C from *Escherichia coli* O7:K1 CE10 (GenBank accession No. CP003037.1). The almost identical RM systems encoded in these plasmids are referred to as SsoII-like RM systems, whereas the corresponding enzymes as SsoII-like enzymes. In other words, the SsoII-like RM systems are spread among various species and genera of the Enterobacteriaceae family.

M.SsoII is a monomer in solution which consists of two domains connected by a flexible linker [26]. The C-terminal domain (residues 72–379) is an MTase. One molecule of M.SsoII recognises the sequence 5′-CCNGG-3′/3′-GGNCC-5′ (N = any nucleotide) in double-stranded DNA and methylates the C5 atom of the inner cytosine residue (Cyt) [27,28,29,30]. The linker (residues 56–71) connects the C-terminal domain to the N-terminal domain (residues 1–55), which acts as a transcription regulator. It binds to the 15-bp palindromic sequence 5′-AGGACAAATTGTCCT-3′/3′-TCCTGTTTAACAGGA-5′ (so-called regulatory site) in the intergenic region of the SsoII RM system, thus repressing transcription of *ssoIIM* gene and stimulating transcription of the *ssoIIR* gene [31,32]. All the specific DNA–protein contacts are located at the regulatory site [33]. Moreover, two M.SsoII molecules bind to one regulatory site [34], and the resulting complex has quite unusual structure: the N-terminal domains are bound to the regulatory site, while the C-terminal domains are bound to DNA flanking the regulatory site [35]. Because the catalytic centre of the MTase domain in such a complex is occupied by non-specific DNA, M.SsoII should lose the ability to bind to the methylation site and to perform DNA methylation. Indeed, a duplex with the regulatory site was shown to inhibit the methylation activity of SsoII-like MTase Ecl18kI [36]. Thus, the two functions of M.SsoII are mutually exclusive.

According to our observations, only low concentrations of M.SsoII exist in a cell. Expression of M.SsoII was shown to delay *E. coli* growth presumably because of its cytotoxicity [37]. An SsoII-like MTase SenPI delayed *E. coli* growth as well perhaps by inducing the SOS response [25].

In a random DNA sequence, the methylation site 5′-CCNGG-3′/3′-GGNCC-5′should be found approximately once every 4^4^ = 256 nucleotides. *S. sonnei* genome comprises 4.8 × 10^6^ bp [38] and therefore should contain ~2 × 10^4^ methylation sites. On the other hand, the 15-bp regulatory site should be found in a random DNA sequence approximately once every 4^15^~10^9^ nucleotides. This number is three orders of magnitude greater than the whole S. sonnei genome. Thus, it is very unlikely to find the regulatory site in the host cell genome. Indeed, a BLAST search against the non-redundant database reveals that nucleotide sequences identical to the regulatory site exist only in SsoII-like RM systems. The cell therefore contains as many regulatory sites as the plasmids encoding the SsoII RM system. The SsoII RM system is encoded in a ColE1-type plasmid [20]. Although there are no empirical data on the copy number of this plasmid, the ColE1-type plasmids in general have copy numbers from a few dozen to several hundred copies per cell [39,40]. Thus, large amount of the methylation sites in a cell contrasts to relatively small amount of the regulatory sites and to low M.SsoII concentration. Therefore, a question arises: what is the mechanism which provides performing both mutually exclusive functions by M.SsoII?

In the present work, we studied in detail the kinetic features of M.SsoII interaction with the methylation site and with the regulatory site. Using the stopped-flow approach, we determined kinetic mechanisms and kinetic parameters of the individual steps of M.SsoII action. By comparing the rate constants of formation of M.SsoII complexes with DNA containing the methylation or regulatory site, we deciphered the mechanism underlying formation of the catalytically competent complex between the enzyme and the methylation site. We demonstrate that such a complex forms efficiently due to the acceleration of the induced fit in the presence of cofactor AdoMet even though M.SsoII prefers the regulatory site at the step of the initial protein–DNA complex formation. We suggest that M.SsoII initially binds AdoMet and remains bound to it in the cell in order to perform the efficient methylation when it finds the target DNA. On the basis of our data, we propose how M.SsoII can successfully implement its two functions in the cell.

## 2. Materials and Methods

All the commercial enzymes and the corresponding buffers used in the present work were acquired from Thermo Fisher Scientific (Waltham, MA, USA).

### 2.1. DNA

**Figure 1:**
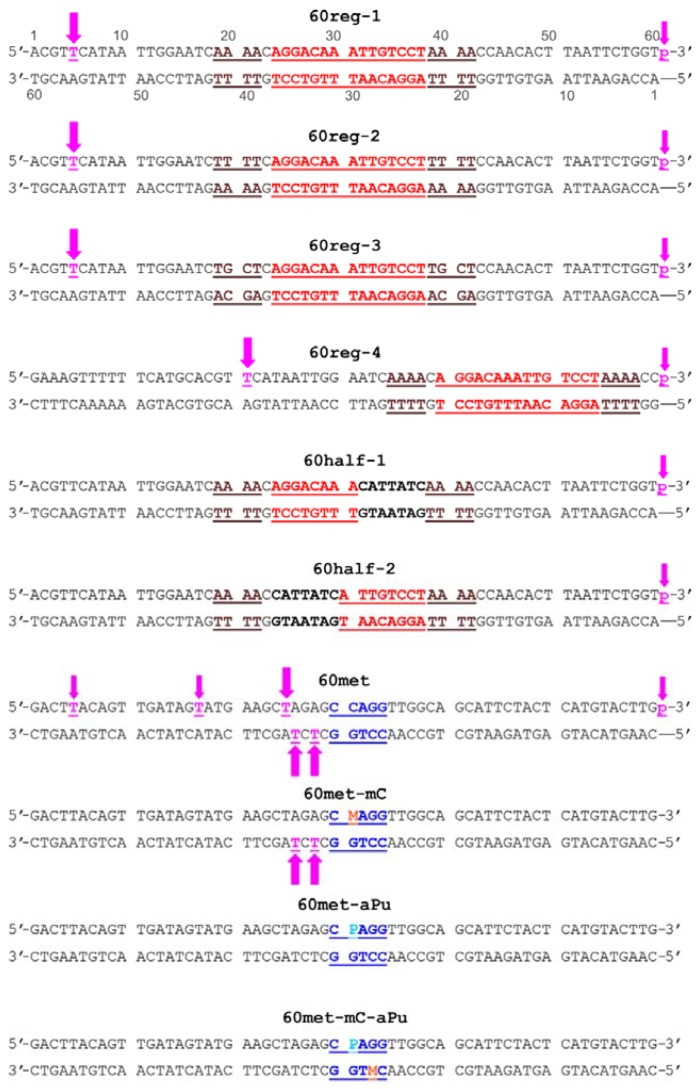
The 60-bp DNA duplexes used in the present work. The methylation site is highlighted in blue, the regulatory site is red, the (A/T)_4_ tracts (or the substituting sequences at the same positions) are brown. The 5-methylcytosine position is marked with an orange letter M. The nucleotide bearing 2-aminopurine is marked with a cyan letter P. Magenta arrows indicate the positions where TAMRA is covalently attached to DNA (each duplex contains only one TAMRA). T stands for deoxyuridine containing TAMRA which was covalently attached to position 5 of the heterocyclic base through a linker; p denotes a phosphate group bearing covalently attached TAMRA. The numbers of nucleotides in ‘top’ and ‘bottom’ strands are indicated for duplex **60reg-1** and are the same for all other duplexes.

Single-stranded DNA fragments were ordered from Syntol (Moscow, Russia), except for 3′-TAMRA-labelled ones, which were kindly provided by Dr. T. Zatsepin (Chemistry Department, Lomonosov Moscow State University, Moscow, Russia). The purity was ‘PAGE grade’ for the DNA fragments with fluorophores and ‘HPLC grade’ for those without fluorophores. Concentrations of single-stranded DNA fragments were measured spectrophotometrically using molar extinction coefficients (ε_260_) determined in the OligoAnalyzer software (http://eu.idtdna.com/analyzer/Applications/OligoAnalyzer). DNA duplexes (Figure 1) were prepared by mixing complementary DNA strands in deionised water, heating them up to 90 °C and cooling them down slowly to room temperature (annealing). The strand without fluorophores was used in a slight excess (5–10%) over that with a fluorophore.

### 2.2. Protein Purification

M.SsoII was expressed from the previously constructed plasmid [37] in *E. coli* M15[pRep4] cells. The recombinant protein carried the following N-terminal His-tag: MRGS(H)_6_TDPLETC. The cells were lysed by sonication in buffer A (20 mM Tris−HCl pH 7.9, 1 М NaCl, 5 mM imidazole, 10% glycerol). M.SsoII was purified by affinity chromatography using Ni-NTA agarose (Qiagen, Hilden, Germany) followed by Heparin Sepharose (GE Healthcare, Chicago, IL, USA), then dialysed against buffer B (50 mM Tris−HCl pH 7.5, 100 mM NaCl, 50% glycerol) and stored at −20 °C.

Traditionally, the influence of a His tag on protein structure is considered negligible, as corroborated by an analysis of crystallized proteins [41]. Indeed, recombinant M.SsoII binds to each one of its recognition sites with high affinity and retains its functions as an MTase and as a transcription factor [31,37]. Moreover, many MTases are known to be co-purified with AdoMet or AdoHcy [42,43,44,45]. The high salt concentration (1 М NaCl) in the lysis buffer ensured thorough purification of M.SsoII from AdoMet, AdoHcy, or any nucleic acids. The absence of AdoMet or AdoHcy was confirmed spectrophotometrically by the A_260_/A_280_ ratio of 0.57. Protein concentrations were measured spectrophotometrically using molar extinction coefficients (ε_280_) determined in the ProtParam software (http://expasy.org/tools/protparam.html). Protein purity was verified by SDS-PAGE; it was ~95%.

### 2.3. Steady-State Analyses of Interaction of M.SsoII with AdoMet and AdoHcy (Trp Fluorescence)

Fluorescence spectra of Trp residues in M.SsoII were registered on a Cary Eclipse Fluorescence Spectrophotometer (Varian, Palo Alto, CA, USA) using medium detector voltage and slow speed at 25 °С. Trp fluorescence was excited at 290 nm (slit of 10 nm) and emission was recorded at 330–380 nm (slit of 10 nm). A quartz cuvette with a 10-mm optical path (Hellma, Müllheim, Germany) was used. M.SsoII concentration in 120 μL of buffer E was 0.8 μM. AdoMet and AdoHcy stock solutions of different concentrations in buffer E were prepared. Their concentrations were determined spectrophotometrically using the molar extinction coefficients ε_260_ = 15,400 M^−1^·cm^−1^ for AdoMet and ε_260_ = 16,000 M^−1^·cm^−1^ for AdoHcy. AdoMet was dissolved immediately prior the experiment and was kept on ice because of its instability. AdoMet or AdoHcy aliquots of 1 μL were added to the protein solution to attain increasing concentrations (from 0.08 to 68 μM). After each addition, the solution was incubated for 2 min before analysis of the spectra. Mixing by pipetting was avoided to prevent oxygenation which could result in quenching of Trp fluorescence [46]. Each spectrum was recorded three times and averaged, followed by subtraction of the initial buffer spectrum. The resulting spectrum was corrected for the protein dilution. Fluorescence intensity at λ_max_ was used to calculate *K*_d_ by means of the modified Stern–Volmer equation: *F*_0_/(*F*_0_ − *F*) = 1/(*f*_a_*K*_SV_[38]) + 1/*f*_a_, where *F*_0_ and *F* are fluorescence intensity values in the absence and presence of the quencher, respectively, *f*_a_ is the fraction of fluorophores accessible to the quencher in the total fluorescence, [38] is the quencher concentration and *K*_SV_ is the quenching constant. In the case when the quenching is caused by formation of a stable complex between the fluorophore and quencher, value *F*_0_/(*F*_0_ − *F*) linearly depends on 1/[38] and does not depend on the fraction of fluorophores inaccessible to the quencher, whereas *K*_SV_ = 1/*K*_d_ [47].

### 2.4. The Electrophoretic Mobility Shift Assay (EMSA)

To estimate M.SsoII affinity to various DNA duplexes, 2.5 nM TAMRA-labelled DNA was titrated with increasing protein concentrations (from 0.75 to 100 nM) in 10 μL of buffer C (50 mM Tris–HCl pH 7.5, 100 mM NaCl, 5 mM β-mercaptoethanol, 0.1 mg/mL BSA, 25% glycerol) containing 100 μM AdoHcy. The presence of AdoHcy in the buffer was required to promote M.SsoII-specific binding to the methylation site in DNA [29], and the same conditions were used in the case of DNA with the regulatory site (for comparison). The reaction mixtures were incubated for 20 min at 37 °C and then cooled to room temperature for 5 min. Each sample was loaded onto a gel in a volume of 10 μL without any additional dyes. Bromophenol blue and/or xylene cyanol served as markers in a marginal lane on a gel. DNA–protein complexes were separated from free DNA by electrophoresis in a flat 20 × 20 × 0.1 cm gel containing a 7% acrylamide:*N,N*’-bis-acrylamide mixture (19:1). Electrophoresis was run in TBE buffer (50 mM Tris–borate, 1 mM EDTA, pH 8.3) at 20 mA and room temperature. TAMRA-containing bands were detected using a Typhoon FLA 9500 scanner (GE Healthcare, USA) with the maximal possible voltage on the photomultiplier. Intensity of the bands was assessed in the ImageQuant software (version 5.0, GE Healthcare).

### 2.5. The Methylation Assay

Efficiency of DNA methylation by M.SsoII was assessed on the basis of the DNA protection from hydrolysis by R.Bme1390I. This enzyme recognises the sequence 5′-CCNGG-3′/3′-GGNCC-5′ (identical to the M.SsoII methylation site) and cleaves it unless it is methylated. The methylation reaction was performed under the same conditions as in the stopped-flow experiments (M.SsoII interaction with TAMRA-labelled duplexes **60met** and **60met-mC** in the presence of 100 μM AdoMet; see below). Two solutions (one with M.SsoII and AdoMet, the other one with TAMRA-labelled DNA and AdoMet) were mixed by hand-pipetting. Aliquots were withdrawn at different time points within 180 s and flash-frozen in liquid nitrogen to stop the reaction. Then, the aliquots were heated up to 90 °С to denature the protein (in case some enzyme molecules retained their activity after the freezing and thawing) and slowly cooled down to room temperature for DNA renaturation. Each aliquot was supplemented with the following reagents: MgCl_2_ up to 10 mM, BSA up to 0.1 μg/μL and R.Bme1390I up to 0.5 U/μL. The reaction mixtures were incubated at 37 °С for 1 h. Proteins were removed from DNA by chloroform extraction, and DNA was analysed by electrophoresis in a non-denaturing 7% polyacrylamide gel (R.Bme1390I produces sticky ends one nucleotide long, and therefore the hydrolysis products are easily separated in a non-denaturing gel, without urea addition). TAMRA-containing bands were detected as described above. To study modification of monomethylated DNA one more step was included in this protocol: an unmethylated DNA strand complementary to the strand that was being methylated was added in 50-fold excess, and reannealing was performed before addition of R.Bme1390I.

### 2.6. Inhibition of Substrate Methylation by Competitor DNA

Two solutions (one containing M.SsoII, the other one containing duplex **60met** (Figure 1) and AdoMet) were mixed by pipetting. The final mixtures contained 4 nM M.SsoII, 10 μM AdoMet and from 8 to 20 nM TAMRA-labelled duplex **60met** in buffer D (50 mM Tris–HCl pH 7.5, 100 mM NaCl, 5 mM β-mercaptoethanol, 0.1 mg/mL BSA). To verify the inhibitory effect of duplex **60reg-1** (Figure 1), different series of reaction mixtures were supplemented with a different amount of **60reg-1** (0, 0.4 or 2 nM). Aliquots were withdrawn at various time points within 30 min and further processed as described above (see the Section 2.5). Initial rates of the methylation reaction were determined for each reaction mixture, and a Lineweaver–Burk plot was constructed to obtain the *K_M_* values.

### 2.7. Plasmid pBend2 Mutagenesis

Plasmid pBend2 [48] was kindly provided by Dr. W. Wende (Justus-Liebig University, Giessen, Germany). Unfortunately, pBend2 contains two instances of the R.SmaI recognition sequence (5′-CCCGGG-3′/3′-GGGCCC-5′). This sequence includes the M.SsoII methylation site (5′-CCNGG-3′/3′-GGNCC-5′) and thus has to be removed from the plasmid before the use in the circular permutation assay. The removal was carried out by means of several rounds of site-directed mutagenesis (Supplementary Information). A mutagenesis scheme with internal and external primers [49] was used for this purpose (Figure S1). The procedure was complicated because both R.SmaI sites were located in an identical sequence context. Each R.SmaI site was changed to 5′-CACGTG-3′/3′-GTGCAC-5′ (see Supplementary Information for details). Sequencing (performed by Evrogen, Moscow, Russia) confirmed the presence of both desirable mutations. The modified plasmid was named pBend2-Mod.

### 2.8. The Circular Permutation Assay

Three short DNA duplexes (each containing one M.SsoII recognition site) were ligated at the R.XbaI and R.SalI sites of the pBend2-Mod plasmid (Figure S2A). The plasmid with the insert served as a template in PCR to obtain a 477-bp fragment (see Supplementary Information for details). This fragment was hydrolysed with a set of REases to obtain a set of linear 162-bp fragments that contained one M.SsoII recognition site (a regulatory or methylation one) at different positions (Figure S2A,B). The 162-bp fragments (5–20 ng, i.e., 10–40 nM) were mixed with M.SsoII (300 nM) in 10 μL of buffer E (50 mM Tris–HCl pH 7.5, 100 mM NaCl, 5 mM β-mercaptoethanol) containing 700 μM AdoHcy, and the mixture was incubated at 37 °C for 30 min. The complexes were separated from free DNA by EMSA on a 7% polyacrylamide gel. The gels were stained with SYBR Gold and photographed with ChemiDoc MS (Bio-Rad, Hercules, CA, USA). Angles of DNA bending were determined using the quadratic equation *y* = *ax^2^* − *bx* + *c*, where *y* is the electrophoretic mobility of the DNA–protein complex (*R_bound_*) normalised to the electrophoretic mobility of the corresponding free DNA (*R_free_*), *x* is flexure displacement (distance between the middle of the protein-binding site and the 5′ end of DNA divided by the full length of the DNA fragment), *a* = −*b* = 2*c*(1 − cosα) and α is the angle of DNA bending [50]. Data from each experiment were fitted independently; fit quality was assessed by means of *R*^2^_adjusted_ (adjusted coefficient of determination)*.* The experiments were repeated at least five times for each set of DNA fragments (with each M.SsoII recognition site).

### 2.9. Stopped-Flow Measurements

Stopped-flow measurements with fluorescence detection were conducted using a SX.20 Stopped-Flow Spectrometer (Applied Photophysics, Leatherhead, UK) equipped with a 150-W Xe arc lamp and 20-μL optical cell. The dead time of the instrument was 1.04 ms. For anisotropy measurements, the light was polarised using calcite prisms for the incident beam and in front of each of the two photomultiplier detectors arranged in a T-configuration. Using TAMRA labelled DNA, the G-factor was measured before each experiment where anisotropy was recorded. It was always ~1. The following excitation and emission wavelengths were used: λ_ex_ = 550 nm and λ_em_ > 580 nm (Lytkarino Optical Glass Factory, Lytkarino, Russia) for TAMRA fluorescence; λ_ex_ = 285 nm and λ_em_ > 320 nm (Schott filter WG-320, Schott, Mainz, Germany) for Trp fluorescence; λ_ex_ = 310 nm and λ_em_ > 370 nm (Corion filter LG-370-F, Franklin, MA, USA) for 2-aPu fluorescence. The experiments were carried out at 25 °C in buffer E (50 mM Tris–HCl pH 7.5, 100 mM NaCl, 5 mM β-mercaptoethanol) for the DNA binding and in buffer F (50 mM Tris–HCl pH 7.5, 100 mM NaCl, 5 mM β-mercaptoethanol, 10% glycerol) for the cofactor binding. An M.SsoII solution in one syringe was rapidly mixed with an equal volume of the substrate solution in the other syringe. In the experiments with cofactor binding, M.SsoII concentration in the reaction chamber after mixing was 1.0 μM, while AdoMet or AdoHcy concentrations varied in the range 0.5–2.0 μM (no DNA in the mixture). In the case of M.SsoII interaction with DNA duplexes (Figure 1), the final DNA concentration was 100 nM, while M.SsoII concentration varied in the range 150–400 nM (for TAMRA-labelled duplexes **60reg-1** and **60reg-2**), 75–250 nM (for TAMRA-labelled **60reg-4**), 150–300 nM (for TAMRA-labelled duplexes **60met** and **60met-mC**) or 75–200 nM (for duplexes **60met-aPu** and **60met-mC-aPu**). M.SsoII interaction with DNA was studied under three conditions: in the presence of 100 μM AdoMet, in the presence of 100 μM AdoHcy or in their absence. In the case of the cofactor presence, the reaction buffer contained the same cofactor concentration in both syringes. The investigation of M.SsoII interaction with DNA duplexes were conducted under the conditions where AdoMet or AdoHcy (100 μM) were present in excess over the enzyme (100–400 nM). Given the equilibrium dissociation constant values *K*_d_ determined from the steady-state fluorescence titration (see Section 3.1), under these conditions, >99% of the enzyme was in complex with the cofactor. Fluorescent traces were recorded until no changes were seen in the experimental curves: during 0.5 s for the interaction of M.SsoII with AdoMet or AdoHcy; during 0.5 s for the M.SsoII interaction with the TAMRA-labelled duplexes containing the regulatory site (**60reg-1**, **60reg-2** and **60reg-4**); during 300 s for the M.SsoII interaction with the TAMRA-labelled duplexes containing the methylation site (**60met** and **60met-mC**; Figure 1); and during 0.25 s for the M.SsoII interaction with the duplexes containing 2-aPu at the methylation site. The split time mode was selected for the long traces (300 s). Each trace shown is the average of ≥4 (for fluorescence intensity measurements) or ≥17 experiments (for anisotropy measurements). Usually, uncertainty lies in the range 2–5%. The reported rate constants represent the mean (with a standard deviation) of such datasets. The DNA fluorescence intensity and anisotropy in the absence of M.Ssoll were constant in time. Besides, the Trp fluorescence intensity of M.Ssoll in the absence of DNA, AdoMet and AdoHcy was also constant in time with the absence of noticeable bleaching during the presented time interval (data not shown).

### 2.10. Kinetic Data Analysis

The kinetic curves were fitted beginning of 1.04 ms, that equals to half-time of first bimolecular reaction or less. Such reaction times permit to determine correctly the values of rate constants. For kinetic curves obtained during analysis of TAMRA or Trp fluorescence intensity and TAMRA anisotropy changes we performed preliminary analysis of the signal curves as a sum of exponents at different concentrations of reactants to estimate the number of equilibrium and non-equilibrium steps as described in [51]. For this purpose we used the OriginPro software (version 8.1, OriginLab Corporation, Northampton, MA, USA). Further, sets of kinetic curves taken at different concentrations of the reactants were simultaneously numerically integrated and fitted using the DynaFit software (version 3, BioKin, Watertown, MA, USA) [52]. The stopped-flow traces were directly fitted by expressing the fluorescence intensity (or fluorescence anisotropy; *F_c_*) at any reaction time point *t* as the sum of the background fluorescence (or anisotropy; *F_b_*) and the fluorescence intensity (or fluorescence anisotropy) of each DNA species:

(1) $$F_{c}=F_{b}+\overset{n}{\underset{i=0}{\sum }}F_{i}\left(t\right)$$

where *F_i_(t)* = *f_i_*[C*_i_*(*t*)], *f_i_* is the coefficient of specific fluorescence (or specific anisotropy) or ‘response factor’ for each discernible DNA conformer and [C*_i_*(*t*)] is the concentration of the conformer at any given time *t* (*i* = 0 denotes free DNA; *i* > 0 denotes enzyme–DNA complexes) [53]. This equation is applicable to anisotropy analysis too because the change of fluorescence intensity was less than 10% during reaction course [54]. The ‘response factors’ *f_i_* resulting from the fits are not used in the determination of the equilibrium constants, but rather provide additional information on the signal variation in different conformations. During the fitting of each kinetic curve series, consecutive spreading of the time slot with simultaneous complication of the kinetic mechanism was performed. The inspection of experimental kinetic traces and theoretical curves corresponding to proposed kinetic schemes was performed by analysis of the residual time courses. As the result, the minimal kinetic scheme is proposed, when the fitted kinetic parameters are not mutually dependent. We believed that the error of curves reproducibility was less than fitting error; therefore, we presented errors from fitting procedure in Tables.

Time slots of 1.5–15 or 2–15 ms in each kinetic curve obtained during detection of 2-aPu fluorescence intensity were fitted separately by one exponent in the OriginPro software. For each series of kinetic curves, a weighted average of the observed kinetic constant ($\bar{k}$) was calculated as follows:

(2) $$\bar{k}=\frac{\sum_{i=1}^{n}\frac{1}{\sigma_{i}^{2}}\cdot k_{i}}{\sum_{i=1}^{n}\frac{1}{\sigma_{i}^{2}}}$$

where *k_i_* is the value of the observed kinetic constant obtained by each kinetic curve fitting, *σ_i_* is the standard deviation of this value and *n* is the number of curves in the kinetic series. The standard deviation of weighted average $\bar{k}$ (s($\bar{k}$)) was calculated as follows:

(3) $$s\left(\bar{k}\right)={\left[\overset{n}{\underset{i=1}{\sum }}\frac{1}{\sigma_{i}^{2}}\right]}^{-\frac{1}{2}}$$

## 3. Results

### 3.1. Cofactor Binding

There are two substrates in the methylation reaction, DNA and cofactor AdoMet. After the methyl group transfer, AdoMet is converted into AdoHcy which remains bound to the enzyme. The AdoHcy release is considered the rate-limiting step of the methylation reaction [55]. Research into the binding of AdoMet and AdoHcy to M.SsoII is required for full characterisation of the enzymatic activity. At first, we tested the binding under steady-state conditions.

A paradigmatic bacterial DNA MTase, M.HhaI, contains Trp41 in the cofactor-binding pocket. Stacking between the Trp41 side chain and the adenine moiety of AdoMet (or AdoHcy) results in quenching of Trp fluorescence [56]. This effect was used to measure *K*_d_ of the M.HhaI complex with the cofactor [55,56,57]. Although we have no crystallographic model of the M.SsoII molecule, sequence comparison (data not shown) predicted Trp102 location at the same position inside the cofactor-binding pocket of M.SsoII as the position of Trp41 in the cofactor-binding pocket of M.HhaI. We titrated 0.8 μM M.SsoII with increasing concentrations of AdoMet or AdoHcy and calculated *K*_d_ values using the modified Stern–Volmer equation (see Section 2). M.SsoII contains three Trp residues that may complicate the analysis. Nevertheless, the modified empirical data were perfectly linearised (*R*^2^_adjusted_ > 0.995; Figure S3). This result suggests that the fluorescence quenching is caused by formation of a stable complex between Trp102 and the cofactor, while the other Trp residues, which are inaccessible for the cofactor, do not influence this dependence [47]. The linearisation yielded *K*_d_ values of 0.57 ± 0.04 μM for the M.SsoII complex with AdoMet and 0.49 ± 0.03 μM for the M.SsoII complex with AdoHcy. Thus, M.SsoII affinities for AdoHcy and AdoMet are comparable.

**Figure 2:**
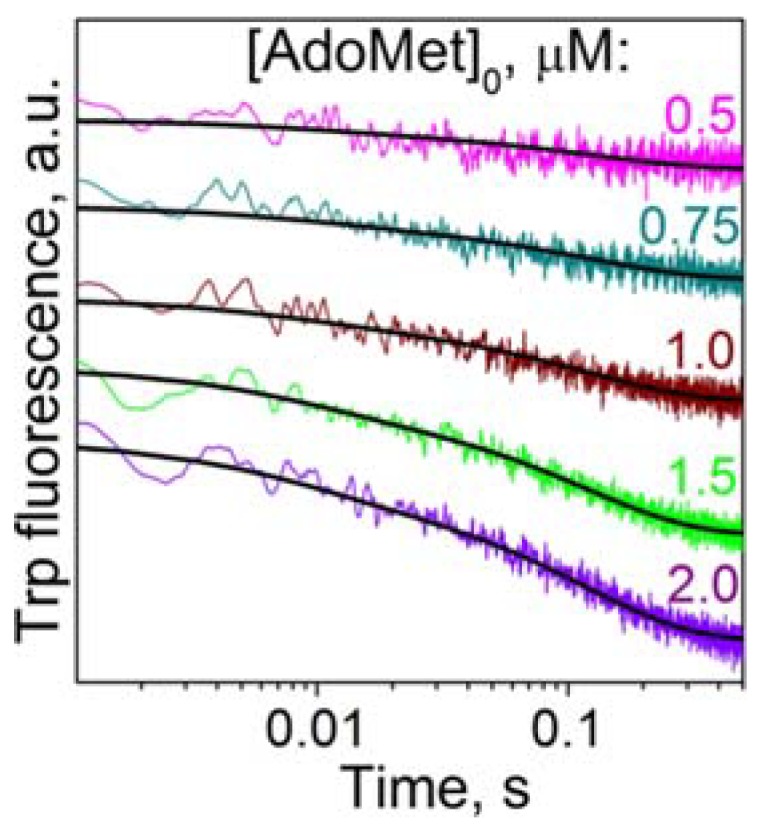
Tryptophan fluorescence traces of M.SsoII binding to AdoMet.

**Scheme 1:**
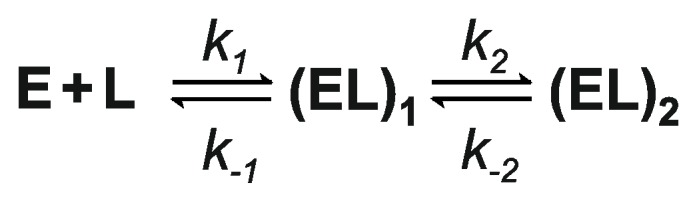
The kinetic mechanism describing the interactions of M.SsoII with the cofactor AdoMet. E is an M.SsoII molecule, L is the AdoMet ligand, (EL)_1_ and (EL)_2_ are enzyme–ligand complexes.

Kinetics of M.SsoII interaction with AdoMet was studied by registering Trp fluorescence on the stopped-flow device (Figure 2). The reaction mixtures contained no DNA. The binding process was fitted to a scheme containing two reversible steps (Scheme 1).

The first step corresponds to the initial enzyme–ligand binding; the second one reflects isomerisation of the complex. The rate constants are summarised in Table 1

The binding affinity of M.SsoII for AdoMet determined from the steady-state fluorescence titration isn’t consistent with the binding affinity that could be calculated from kinetic parameters summarised in Table 1. These data can be explained as follows: probably the additional isomerisation step (or steps) of the enzyme–ligand complex that we have not registered exists during the interaction of M.SsoII with AdoMet. Probably we have not registered this additional step due to Trp bleaching at time points >10 s. Consequently, we have not obtained the complete set of rate constants for M.SsoII interaction with AdoMet for correct calculation of equilibrium association constant. Therefore, during the comparison of M.SsoII affinities for AdoHcy and AdoMet we rely upon equilibrium dissociation constant values *K*_d_ determined from the steady-state fluorescence titration. We use rate constants for M.SsoII interaction with AdoMet only for the comparison of M.SsoII initial binding to cofactor and target DNA.

### 3.2. Design of the DNA Duplexes

To analyse the interaction of M.SsoII with the regulatory site, one needs a sufficiently long DNA molecule because of the following empirical findings. Firstly, DNase I footprinting showed that M.SsoII protected 48 nucleotides from hydrolysis in one DNA strand and 52 nucleotides in the other one [31]. Secondly, chemical cross-linking data allowed us to build a model of the M.SsoII complex with the regulatory site where long DNA was absolutely necessary for the proper structure of the complex [35]. Thus, we used 60-bp DNA duplexes in the present study (Figure 1). Some of these duplexes were successfully utilised in our previous experiments with M.SsoII [34,35,58].

The main DNA duplex is **60reg-1**; it contains the regulatory site (red in Figure 1) at the centre, while the flanking sequences are exactly as they are in the naturally occurring plasmid that encodes the SsoII RM system. Efficiency of the interaction between a DNA-binding protein and its target DNA is known to also depend on the sequence flanking the target site [59]. The quasipalindrome regulatory site with central A/T pare is flanked by 5′-AAAA-3′/3′-TTTT-5′ sequences (or (A/T)_4_ tracts; green in Figure 1) on both sides. A/T tracts are well known to induce DNA bending [60]. To test the role of such tracts in formation of the complex between M.SsoII and the regulatory site, we used DNA duplexes **60reg-2** and **60reg-3**. In **60reg-2**, the (A/T)_4_ tracts were changed to (T/A)_4_ tracts (5′-TTTT-3′/3′-AAAA-5′). **60reg-3** is a variant with a random sequence (5′-TGCT-3′/3′-TCGT-5′) instead of the (A/T)_4_ tracts (see Figure 1). To confirm the importance of DNA length for the complex formation, we used duplex **60reg-4**, where the regulatory site was shifted towards one of the duplex ends. Duplexes **60half-1** and **60half-2** contain either the ‘left’ or ‘right’ half of the regulatory site and are supposed to bind to only one M.SsoII subunit. Finally, in order to compare the interaction of M.SsoII and the regulatory site versus the methylation site, we used duplexes of the same length containing the methylation site (blue in Figure 1) at the centre, namely **60met** (not methylated) and **60met-mC** (methylated in the ‘top’ strand).

To detect DNA and DNA–protein complexes in EMSA and stopped-flow experiments, we used fluorescently labelled DNA. 5-[and-6]-Carboxytetramethylrhodamine (TAMRA) was chosen as a fluorophore because it is fairly stable chemically, relatively cheap and has a sufficient quantum yield for detection at desirable DNA concentrations. Only one TAMRA dye was coupled to each duplex. Different variants of TAMRA positioning were tested (magenta arrows in Figure 1). In the first variant, TAMRA was covalently attached to position 5 of a thymine through a linker. The modified nucleotide was located inside the duplex (at least 4 bp away from the duplex end). In the second variant, TAMRA was covalently attached to the phosphate group of the 3′-terminal nucleotide. It showed significantly lower fluorescence intensity (presumably, a decreased quantum yield because of fluorophore stacking to the 3′-end base pair), which required higher DNA concentrations in EMSA experiments. Thus, the first variant of TAMRA positioning was preferred for most of DNA duplexes.

When a protein binds to a fluorescently labelled DNA, it restricts the mobility of the fluorophore to some extent thereby increasing fluorescence anisotropy. We planned to use this effect to detect DNA–protein complex formation on the stopped-flow device. Furthermore, TAMRA in some locations (positions 5 and 17 of the ‘top’ strand in duplex **60met**; see small magenta arrows in Figure 1) did not yield an anisotropy increase upon M.SsoII binding to the corresponding duplex. Probably the fluorophore at these positions was too far from the protein molecule bound to the methylation site. TAMRA at other locations showed the desirable anisotropy increase upon the M.SsoII binding (namely, position 25 in the ‘top’ strand of duplex **60met**, positions 33 and 35 in the ‘bottom’ strand of duplexes **60met** and **60met-mC**, position 5 in duplexes **60reg-1**, **60reg-2** and **60reg-3** as well as position 21 in duplex **60reg-4**; see large magenta arrows in Figure 1). These duplexes were used in our further experiments. DNA duplexes of the same sequence but with different TAMRA positioning bound to M.SsoII with similar affinity (data not shown).

### 3.3. Formation of a Complex between M.SsoII and Various DNA Duplexes

**Figure 3:**
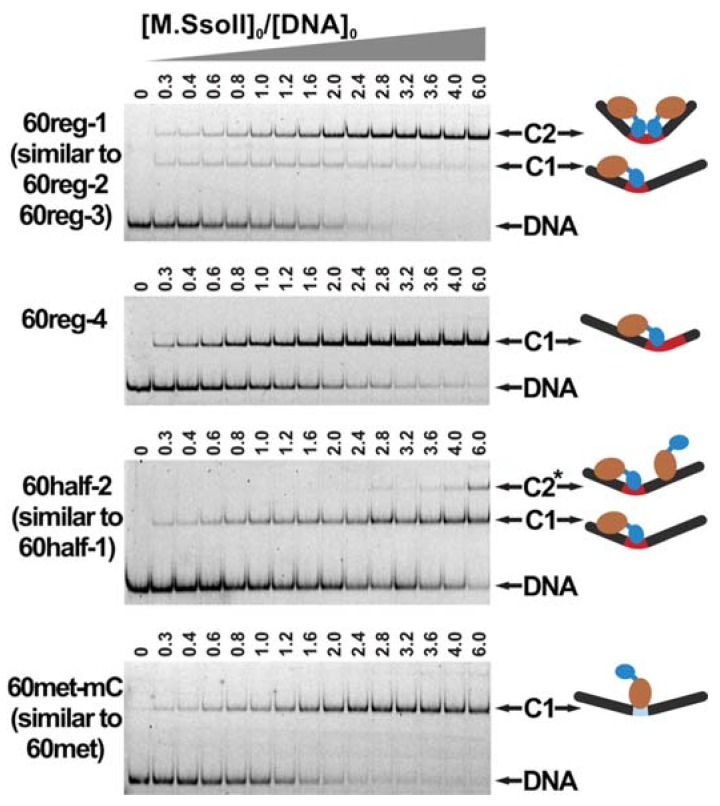
Formation of a complex between M.SsoII and various TAMRA-labelled 60-bp DNA duplexes (indicated on the left) analysed by EMSA in a non-denaturing 7% polyacrylamide gel. The ratio of M.SsoII initial concentration to DNA initial concentration is shown above the lanes. DNA concentration is 2.5 nM, M.SsoII concentration varies from 0.75 to 15 nM. Complex **C1** consists of one M.SsoII subunit and one DNA duplex. Complex **C2** consists of two M.SsoII subunits bound to the regulatory site and one DNA duplex. Complex C2* presumably contains one DNA duplex, one M.SsoII subunit bound specifically to the regulatory half-site and the other M.SsoII molecule bound to the flanking sequence in a non-specific manner. The overall results of complex formation are qualitatively similar for M.SsoII complexes with **60reg-1**, **60reg-2** and **60reg-3**; with **60half-1** and **60half-2**; or with **60met-mC** and **60met** (indicated in brackets; results for those DNA duplexes which are omitted here are shown in Figure S4). Structures of the complexes are presented schematically on the right taking into account the angles of DNA bending determined in this work (see below). DNA is black, the regulatory site is red, and the methylation site is light blue. The N-terminal domain of M.SsoII is blue; the C-terminal domain is brown.

Complex formation was carried out at low concentrations: 2.5 nM DNA was titrated with increasing M.SsoII amounts (from 0.75 to 100 nM) in the presence of 100 μM AdoHcy. The products of M.SsoII binding to different 60-bp DNA duplexes were analysed by EMSA (Figure 3 and Figure S4). Only one DNA–protein complex was detected upon M.SsoII interaction with **60met** or **60met-mC** at up to sixfold protein excess over DNA, while two complexes were seen upon M.SsoII interaction with **60reg-1**, **60reg-2** or **60reg-3**, in agreement with our previous data [34,35]. This result indicates that only one M.SsoII molecule binds to the methylation site, while the regulatory site can bind to one or two M.SsoII subunits (marked as complexes **C1** and **C2** in Figure 3). Remarkably, M.SsoII binding to the regulatory site is highly cooperative: there is always much more complex **C2** than complex **C1** (Figure 3 and Figure S4). We found no conditions under which complex **C1** would exist without any admixture of complex **C2**.

In order to obtain only complex **C1**, we tried duplexes **60half-1** and **60half-2**, each containing a half of the regulatory site. Unexpectedly, complex C2* with a lower electrophoretic mobility appeared when M.SsoII/DNA ratio exceeded 2.0 and became prominent at the fourfold excess, when a significant fraction of DNA was not yet bound to the protein (Figure 3 and Figure S4). We assume that in complex C2*, one M.SsoII molecule was bound to the half of the regulatory site, while the flanking DNA sequences provided enough space for non-specific binding of the second M.SsoII molecule. Thus, such duplexes were not suitable for research on M.SsoII-specific complexes with its target sites and therefore were not used in any further experiments.

Complex **C1** without complex **C2** was formed in the case of **60reg-4**. It is possible that one M.SsoII molecule was bound normally to the ‘left’ half of the regulatory site, while the lack of a long flanking sequence ‘to the right’ of the regulatory site heavily impaired binding of the second M.SsoII molecule (shown schematically in Figure 3). Hence, duplex **60reg-4** was further utilised to investigate formation of complex **C1**.

### 3.4. DNA Bending by M.SsoII

Many transcription factors are known to bend their target DNA, including C proteins, which regulate gene expression in numerous RM systems [61,62]. Some DNA MTases also induce bending in their target DNA [63,64,65,66]. To test the ability of M.SsoII to bend DNA, we used the classical circular permutation analysis based on plasmid pBend2 [48]. Each M.SsoII recognition site (regulatory or methylation) was introduced into the mutated plasmid (see Materials and Methods and Supplementary Information). The resulting plasmids were hydrolysed with different REases in order to create 162-bp DNA fragments that contained the M.SsoII recognition site at different positions. Three sets of 162-bp fragments were obtained, each set consisting of 11 fragments. The sets were named after the analogous 60-bp duplexes: **162reg-1** (contains the regulatory site with the native flanking sequences: (A/T)_4_ tracts), **162reg-2** (contains the regulatory site with the inverted flanking sequences: (T/A)_4_ tracts) and **162met** (contains the methylation site). Within each set, M.SsoII complexes with the DNA fragments had different electrophoretic mobility patterns depending on the distance from the DNA end to the recognition site.

**Figure 4:**
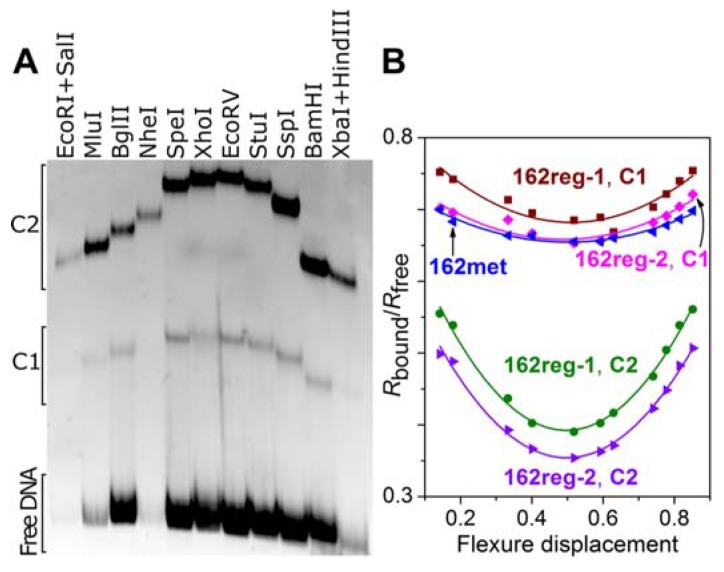
Determination of angles of DNA bending induced by the M.SsoII binding to its recognition sites. (**A**) Electrophoretic mobility data on M.SsoII complexes with 162-bp DNA fragments that carry the regulatory site at different positions (the set **162reg-1** is shown) depending on the distance from the DNA 5′-end to the recognition site. The REases that were used to obtain each DNA fragment are indicated above the corresponding lanes. EMSA in a non-denaturing 7% polyacrylamide gel followed by SYBR Gold staining; (**B**) Electrophoretic mobility of each DNA–protein complex (*R*_bound_) normalised to the electrophoretic mobility of the corresponding free DNA (*R*_free_) as a function of flexure displacement (the distance between the bending site and the DNA 5′-end). Experimental data on M.SsoII binding to DNA fragments **162reg-1**, **162reg-2** and **162met** are fitted to a quadratic function.

After formation of the M.SsoII complex with the sets of DNA fragments containing the regulatory site (**162reg-1** and **162reg-2**), two types of DNA–protein complexes formed (**С1** and **С2**, see above). There was much more complex **C2** than complex **C1**, just as in Figure 3, pointing to high cooperativity of M.SsoII binding to the regulatory site in accordance with our earlier data when M.SsoII was cross-linked to DNA duplexes containing a reactive group at the regulatory site [67]. Complexes **C2** showed significant differences in the electrophoretic mobility depending on the position of the regulatory site (Figure 4A). These empirical data were used to calculate angles of DNA bending (Figure 4B). The data obtained for complexes **C2** were fitted with high accuracy, judging by the adjusted coefficient of determination (*R*^2^_adjusted_) in Table 2. Differences in the electrophoretic mobility among complexes **C1** were more moderate, indicating a smaller angle. Accordingly, the accuracy of fitting was lower for complexes **C1** because circular permutation analysis is known to have high accuracy for large angles [68].

The results for the regulatory site flanked by (A/T)_4_ or (T/A)_4_ tracts were equal within the margin of error: DNA showed a bend of ~90° in complex with the M.SsoII dimer and ~45° in complex with the M.SsoII monomer (Table 2)*.* Thus, M.SsoII played a key role in formation of the bend, whereas the (A/T)_4_ tracts barely influenced the DNA bending. The M.SsoII dimer induced an angle twice as large as the M.SsoII monomer did when bound to the regulatory site. In other words, both M.SsoII subunits made equal contributions in the resulting DNA bending; both monomer-induced angles lied in the same plane. The N-terminal domain of M.SsoII shares a high similarity with C proteins [34], which also induce large angles in their target DNA [61].

After formation of the M.SsoII complex with the set of DNA fragments containing the methylation site (**162met**), only one type of DNA–protein complexes (**C1**) was detected, and the electrophoretic mobility varied moderately (data not shown), as was the case for complexes **C1** formed with the regulatory site. The bend of ~31° induced by M.SsoII at the methylation site is over the range shown for other C5-MTases by circular permutation analysis, being most similar to the bend induced by M.HpaII [65,66].

### 3.5. DNA with the Regulatory Site Inhibits the MTase Activity

Because M.SsoII has two different DNA-binding sites located in two different domains, one can imagine a complex where an M.SsoII molecule would be bound simultaneously to the methylation site and to the regulatory site. Although such a complex was fixed in our previous work [67], where two synthetic DNA duplexes with reactive groups were covalently cross-linked to the protein, we have never detected this complex experimentally under physiological conditions. Moreover, we found that the presence of **60reg-1** in a reaction mixture significantly decreased the binding of M.SsoII to **60met** in the presence of cofactor analogue AdoHcy (data not shown). Furthermore, it was reported that a duplex with the regulatory site inhibits enzymatic activity of the SsoII-like MTase Ecl18kI [36]. In those experiments, a 31-bp duplex with the regulatory site was used in 20-fold to 200-fold excess over the protein to demonstrate the inhibitory effect. We performed similar experiments, where **60reg-1** inhibited M.SsoII activity. Such a long duplex was a very potent inhibitor and its effect was prominent already at the **60reg-1**/M.SsoII ratio of 0.5 (Figure S6): although methylation of **60met** by M.SsoII was characterised by apparent *K*_M_ = 1.6 nM in the absence of **60reg-1**, the apparent *K*_M_ value increased up to 10 nM at the **60reg-1**/M.SsoII ratio of 0.5. Considering such a potent inhibitory action of the regulatory site, one could assume that M.SsoII is a weakly active MTase in the cell. To check this, we studied in detail the kinetics of M.SsoII interaction with each one of its binding sites.

### 3.6. Kinetics of M.SsoII Binding to DNA with the Regulatory Site

**Figure 5:**
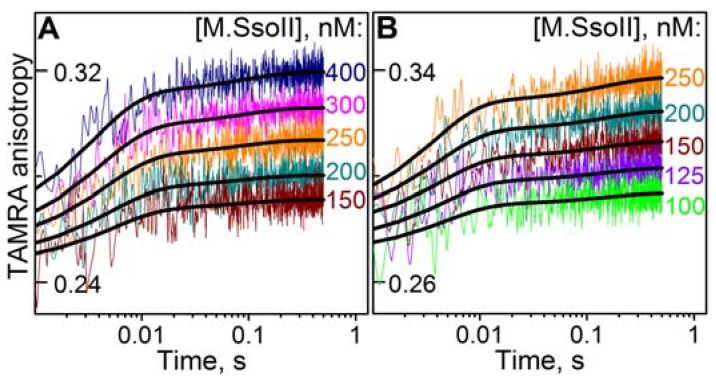
TAMRA anisotropy traces of duplex **60reg-1** (**A**) or **60reg-4** (**B**) during their binding with M.SsoII in the presence of AdoMet (100 μM). Smooth curves are the result of the fitting procedure. Concentrations of substrates are 100 nM. Concentrations of M.SsoII are indicated next to each curve. Note: the time scale is logarithmic here and in Figure 5, Figure 6, Figure 7, Figure 8, Figure 9 and Figure 10.

M.SsoII interaction with the regulatory site implies the DNA–protein complex formation without any chemical reaction. To monitor this process, we placed TAMRA in the DNA duplex (Figure 1) and registered its anisotropy changes upon the M.SsoII binding using stopped-flow technique under single-binding conditions. Representative TAMRA anisotropy traces obtained at increasing M.SsoII concentrations are shown in Figure 5. Despite quite a long distance (18 bp) between the regulatory site and the fluorophore, we obtained the desired increase in anisotropy which confirmed some restrictions of TAMRA mobility caused by formation of the DNA–protein complex.

Most stopped-flow experiments are typically performed at DNA and protein concentrations approximately 10^−6^ or 10^−5^ M. The binding between M.SsoII and its target DNA was found to be so rapid that we were forced to decrease the concentrations to 10^−7^ M (100 nM) to slow down the process and make it measurable. Lowering the concentration of TAMRA-labelled DNA resulted in heavily increased experimental noise. Nevertheless, averaging the signals of multiple identical shots allowed us to reduce the noise and to decipher the kinetics of the process.

Although transcriptional regulation by M.SsoII should not depend on the cofactor presence or absence, this protein is likely to be bound to AdoMet or AdoHcy in the cell. Therefore, we examined the kinetics of M.SsoII interaction with the regulatory site in all possible combinations (with AdoMet, with AdoHcy or without any of them).

**Scheme 2:**
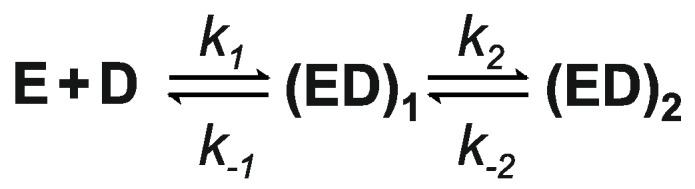
The kinetic scheme describing TAMRA anisotropy traces of duplexes containing the regulatory site (regardless of the cofactor presence) or the methylation site (in the presence of AdoHcy) upon their interactions with M.SsoII. E is an M.SsoII molecule, D is the DNA duplex, while (ED)_1_ and (ED)_2_ are DNA–protein complexes.

Fitting the empirical data in DynaFit (Figure 5 and Table 3) yielded a two-stage kinetic scheme for M.SsoII binding to any duplex containing the regulatory site (**60reg-1** or **60reg-2**; Scheme 2).

It is tempting to assume that the first stage represents the binding between the DNA and one M.SsoII molecule and to interpret the second stage as the binding of the second M.SsoII subunit to the complex. In contrast, a control experiment with **60reg-4** disproved this hypothesis. Although only one M.SsoII molecule binds to **60reg-4**, the interaction consists of two similar stages. Therefore, the first stage represents M.SsoII binding to the DNA, whereas the second stage should be interpreted as a conformational change in the complex. We can theorise that this solitary conformational change is associated with the DNA bending (Table 2). Thus, our experimental approach does not detect the binding of the second M.SsoII molecule to **60reg-1** or **60reg-2** because each DNA duplex contains only one fluorophore, and this fluorophore is accessible to one of the two M.SsoII subunits.

No considerable differences were observed in the values of the corresponding rate constants for M.SsoII monomer interaction with duplexes **60reg-1** and **60reg-2**, which contain (A/T)_4_ and (T/A)_4_ tracts, respectively (Table 3). Therefore, we can say that DNA bending induced by the (A/T)_4_ tracts flanking the regulatory site barely influences the kinetic parameters of the initial binding of M.SsoII and the subsequent conformational rearrangement in the enzyme–DNA complex.

### 3.7. Kinetics of M.SsoII Interaction with DNA Containing the Methylation Site

Next, we focused on the kinetic mechanism of M.SsoII interaction with the methylation site. Besides, we aimed at finding differences in the kinetics of M.SsoII interaction with the methylation site and with the regulatory site. The DNA duplex with the methylation site was relatively long (60 bp) for the purpose of comparison with other 60-bp duplexes, although typically, short DNA duplexes (12–25 bp) are used in studies on methylation. One disadvantage of the long DNA was the inability to detect target cytosine flipping and covalent-bond formation directly by UV absorbance at 280 nm (as in [67]). Therefore, we used duplexes with TAMRA placed near the methylation site in order to detect DNA–protein complex formation and its further conformational changes and duplexes with a 2-aminopurine (2-aPu) residue inside the methylation site in order to study the target base flipping.

One of the TAMRA-labelled DNA duplexes (**60met**) contained two cytosine residues that could be methylated by M.SsoII. The other TAMRA-labelled duplex (**60met-mC**) contained one such cytosine residue because the second one was preliminarily methylated. DNA methylation depends on the cofactor presence [3]. Therefore, we examined the kinetics of M.SsoII interaction with the methylation site in the presence of either the cofactor AdoMet or its inactive analogue AdoHcy.

To prove the possibility of methylation under the conditions chosen for the stopped-flow experiments, we mixed the same components by hand-pipetting and analysed the reaction products by hydrolysis with R.Bme1390I, which does not cleave mono- and dimethylated sites 5′-CCNGG-3′/3′-GGNCC-5′ (Figure S7). The first time-point taken by hand-pipetting was 10 s when ≥40% of DNA was already converted into the reaction product, while at 100 s, the amount of the product reached a plateau.

#### 3.7.1. Interaction of M.SsoII with TAMRA-Labelled DNA Containing the Methylation Site in the Presence of AdoHcy

**Figure 6:**
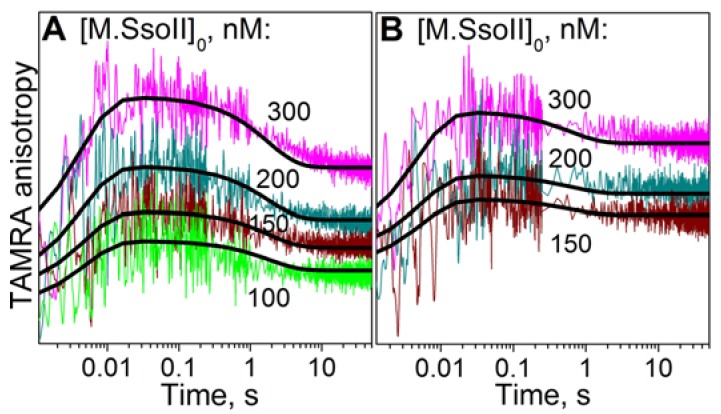
TAMRA anisotropy traces of substrate **60met-mC** (**A**) or **60met** (**B**) during their binding with M.SsoII in the presence of AdoHcy (100 μM). Smooth curves are the result of the fitting procedure. Concentrations of substrates are 100 nM. Concentrations of M.SsoII are indicated next to each curve.

Figure 6 depicts the kinetic traces obtained by detecting changes in TAMRA anisotropy for the M.SsoII interaction with **60met-mC** or **60met** in the presence of AdoHcy. Initially, an increase in TAMRA anisotropy occurred during the time interval 0 ms to 20 ms in both kinetic series. The anisotropy then showed a slow decrease. The increase likely reflected the formation of an initial enzyme–substrate complex. It was hypothesised that the subsequent decrease in the anisotropy corresponded to isomerisation of the initial complex to produce the second DNA–protein complex. The substrate underwent conformational adjustment during this process. Such double-stage interaction was described using a kinetic scheme containing two reversible steps, which was identical to Scheme 2.

Fitting the empirical data in DynaFit (Figure 6) according to this scheme provided the rate constants of the M.SsoII interaction with each duplex with the methylation site (**60met-mC** or **60met**) in the presence of AdoHcy (Table 4). The values of the corresponding rate constants of the initial M.SsoII complex formation with **60met-mC** or **60met** (*k*_1_, *k*_−1_) are comparable. These data revealed that the number of unmethylated Cyt within the methylation site has no significant influence on the initial binding. On the other hand, the number of unmethylated Cyt has an influence on the subsequent isomerisation of the enzyme–substrate complex because the values of the corresponding rate constants *k*_2_* and *k*_−2_* differ for **60met-mC** and **60met**.

**Figure 7:**
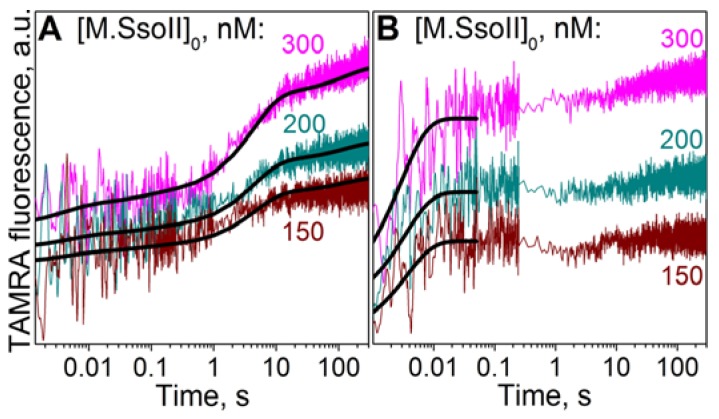
TAMRA fluorescence traces of substrates **60met-mC** (**A**) or **60met** (**B**) during their binding with M.SsoII in the presence of AdoHcy (100 μM). Smooth curves are the result of the fitting procedure. Concentrations of substrates are 100 nM. Concentrations of M.SsoII are indicated next to each curve.

The kinetic traces obtained during the M.SsoII interaction with the methylation site (**60met-mC** or **60met**) in the presence of AdoHcy, with detection of changes in TAMRA fluorescence intensity are shown in Figure 7. An increase in the fluorescence intensity occurred in the kinetic series for both duplexes. The initial increase in the time interval 0 ms to 20 ms likely reflected formation of an initial enzyme–substrate complex. The subsequent increase in the fluorescence intensity probably corresponded to the isomerisation of the initial complex.

**Scheme 3:**
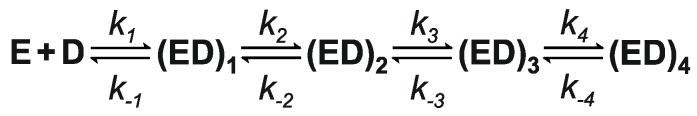
The kinetic scheme describing TAMRA fluorescence traces of substrate **60met-mC** during its interaction with M.SsoII in the presence of AdoHcy. E is an M.SsoII molecule, D is a DNA duplex, while (ED)_1_, (ED)_2_, (ED)_3_ and (ED)_4_ are DNA–protein complexes.

Fitting the empirical data (Figure 7A and Table 4) yielded a four-stage kinetic scheme for the M.SsoII interaction with substrate **60met-mC** in the presence of AdoHcy (Scheme 3).

This result suggested that the formation of the initial enzyme–substrate complex was followed by three isomerisation steps. Thus, the analysis of changes in TAMRA fluorescence intensity allowed us to uncover two additional steps of enzyme–substrate complex isomerisation that were not observed during the monitoring of anisotropy changes. When analysing the fluorescence kinetic traces of M.SsoII interaction with **60met-mC** (Figure 7A), we assumed the kinetic constants of the initial enzyme–substrate complex formation (*k*_1_, *k*_−1_) to be equal to the corresponding rate constants obtained during fitting of the kinetic traces of anisotropy (Figure 6A). The negligible amplitude of the fluorescence intensity changes in the time interval 0 ms to 20 ms did not allow us to fit these rate constants directly from the fluorescence kinetic traces (Figure 7A).

The changes in fluorescence intensity at the time points beyond 20 ms were less pronounced in the case of substrate **60met** (Figure 7B) than those for substrate **60met-mC**. The kinetic traces of M.SsoII interaction with **60met** allowed us to fit only the rate constants of the first reversible step, corresponding to the initial complex formation (Table 4).

#### 3.7.2. Interaction of M.SsoII with TAMRA-Labelled DNA Containing the Methylation Site in the Presence of AdoMet

M.SsoII interaction with the methylation site in the presence of AdoMet leads to formation of a catalytically competent DNA–protein complex and to subsequent methylation of the target cytosine residues. To monitor this process, the conformational dynamics of TAMRA-labelled DNA **60met-mC** or **60met** upon its interaction with M.SsoII in the presence of AdoMet was analysed via detection of changes in TAMRA fluorescence intensity and anisotropy.

**Figure 8:**
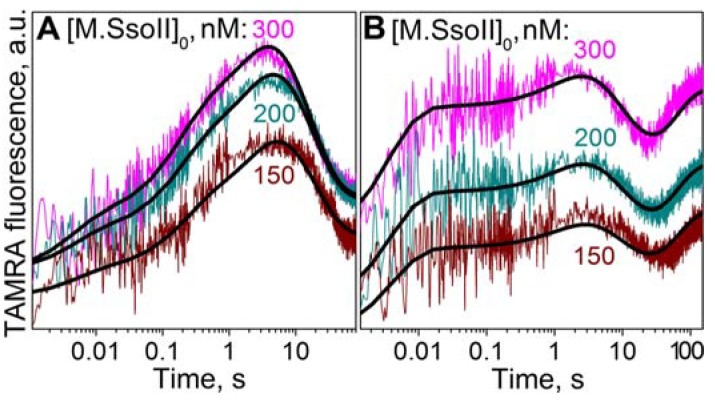
TAMRA fluorescence traces of substrate **60met-mC** (**A**) or **60met** (**B**) during their interaction with M.SsoII in the presence of AdoMet (100 μM). Smooth curves are the result of the fitting procedure. Concentrations of substrates are 100 nM. Concentrations of M.SsoII are indicated next to each curve.

The kinetic traces obtained for the changes in TAMRA fluorescence intensity are presented in Figure 8. In the case of substrate **60met-mC** (Figure 8A), the initial multiphase increase in the fluorescence intensity lasted for 4–5 s. This increase likely corresponded to formation of an initial enzyme–substrate complex and its isomerisation. After that, the fluorescence intensity was decreasing (Figure 8A). Kinetic curves of M.SsoII interaction with **60met-mC** in the presence of AdoHcy lacked such a decrease in fluorescence intensity (Figure 7A). According to these data, we can say that the fluorescence intensity decrease in Figure 8A corresponds to formation of the catalytically competent complex leading to methylation of Cyt in the substrate. At the same time, TAMRA fluorescence intensity was still increasing at the time points beyond 4–5 s in the kinetic traces during M.SsoII interaction with **60met-mC** in the presence of the cofactor analogue (Figure 7A). This increase corresponded to isomerisation of the enzyme–substrate complex. On the basis of these data, we believe that the enzyme ‘is trying’ to carry out the methylation reaction changing its conformation even under the conditions that prevent substrate methylation, i.e., in the presence of AdoHcy.

The interaction of M.SsoII with **60met** in the presence of AdoMet was accompanied by an initial increase in TAMRA fluorescence intensity for 2–3 s likely corresponding to formation of an initial enzyme–substrate complex and its isomerisation (Figure 8B). The fluorescence intensity then showed a decrease lasting from 2–3 s to 20–30 s. This decrease was consistent with the fluorescence intensity decrease observed in the kinetic traces of **60met-mC** during its interaction with M.SsoII at the time points beyond 4–5 s (Figure 8A). Given these data, the fluorescence intensity decrease from 2–3 s to 20–30 s (Figure 8B) most likely corresponds to formation of the catalytically competent complex leading to methylation of one Cyt in substrate **60met**. The level of fluorescence intensity did not return to its initial value, indicating that M.SsoII did not release the monomethylated substrate. TAMRA fluorescence intensity then showed an increase, and the traces entered a plateau phase (Figure 8B). The fluorescence intensity increase at the time points after 20–30 s likely corresponds to formation of the catalytically competent complex leading to methylation of the other Cyt. Final levels of TAMRA fluorescence intensity did not return to their initial values in both kinetic series (Figure 8). This finding indicated that the terminal dissociation step was not reflected in the fluorescence kinetic curves of the M.SsoII interaction with the methylation site in the presence of AdoMet. Thus, the complexes of the enzyme with monomethylated or dimethylated substrates are rather stable.

**Scheme 4:**
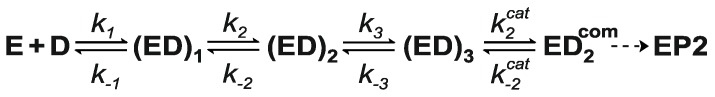
The kinetic scheme describing the interactions of M.SsoII with substrate **60met-mC** in the presence of AdoMet. E is an M.SsoII molecule, D is a DNA substrate, (ED)_1_, (ED)_2_ and (ED)_3_ are enzyme–substrate complexes, ${\mathrm{ED}}_{2}^{\mathrm{com}}$ is a catalytically competent complex, whereas EP2 is the complex of the enzyme with a methylation reaction product (dimethylated DNA duplex).

The M.SsoII interactions with substrate **60met-mC** in the presence of AdoMet (Figure 8A) were described by means of a kinetic scheme containing four steps (Scheme 4). This scheme included a step of formation of the initial enzyme–substrate complex, and two steps of its isomerisation, followed by a step of formation of the catalytically competent complex leading to the cytosine residue methylation.

We were not able to differentiate between the formation of the catalytically competent complex and the methylation reaction of the cytosine residue. Such step (here and further) was fitted to a reversible equilibrium; therefore, the formation of the catalytically competent complex limits the rate of this step. The methyl transfer reaction itself is irreversible (see Section 4.1.2. for the explanation), but we could not determine the value of its rate constant.

**Scheme 5:**
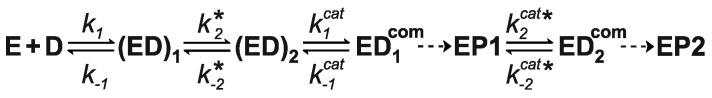
The kinetic scheme describing TAMRA fluorescence traces of substrate **60met** during its interaction with M.SsoII in the presence of AdoMet. E is an M.SsoII molecule, D is a DNA substrate, (ED)_1_ and (ED)_2_ are enzyme–substrate complexes, ${\mathrm{ED}}_{1}^{\mathrm{com}}$ and ${\mathrm{ED}}_{2}^{\mathrm{com}}$ are catalytically competent complexes, whereas EP1 and EP2 are the M.SsoII complexes with the methylation reaction products: mono- and dimethylated duplexes, respectively.

Fitting the fluorescence traces of **60met** during its interaction with M.SsoII in the presence of AdoMet (Figure 8B) also yielded a kinetic scheme containing four steps (Scheme 5). In this case, the step of formation of the initial enzyme–substrate complex was followed by one step of its isomerisation, a step of formation of the catalytically competent complex leading to methylation of the ‘first’ cytosine residue and a step of formation of the catalytically competent complex leading to methylation of the ‘second’ cytosine residue.

Thus, the kinetic traces presented in Figure 8B did not allow us to distinguish the two steps of isomerisation of the enzyme–substrate complex. Fitting of the fluorescence traces provided the rate constants of interaction of M.SsoII with **60met-mC** or **60met** in the presence of AdoMet (Table 4). Rate constants *k*_2_* and *k_−_*_2_* are not the absolute kinetic parameters but rather effective ones describing the joint process of isomerisation. Probably for this reason, the kinetic constant *k_−_*_2_* was determined with a large error (Table 4).

**Figure 9:**
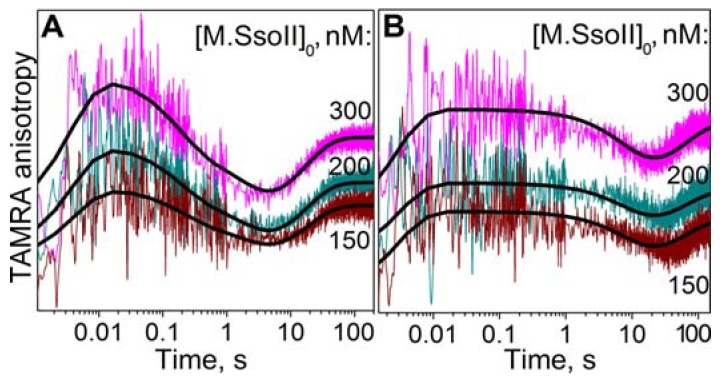
TAMRA anisotropy traces of substrate **60met-mC** (**A**) or **60met** (**B**) during their interaction with M.SsoII in the presence of AdoMet (100 μM). Smooth curves are the result of the fitting procedure. Concentrations of substrates are 100 nM. Concentrations of M.SsoII are indicated next to each curve.

Figure 9 represents the kinetic traces obtained during the M.SsoII interaction with **60met-mC** or **60met** in the presence of AdoMet, with detection of TAMRA anisotropy changes. Initially, an increase in TAMRA anisotropy occurred during the time interval 0 ms to 20 ms in both kinetic series. This increase likely corresponded to formation of the initial enzyme–substrate complex.

In the case of substrate **60met-mC** (Figure 9A), the anisotropy then showed a decrease lasting up to 4–5 s, which likely reflected isomerisation of the initial complex. TAMRA anisotropy then was increased, reaching a plateau (Figure 9A). This anisotropy increase coincided with the fluorescence intensity decrease at the time points beyond 4–5 s in the kinetic traces presented in Figure 8A. Besides, kinetic traces of the M.SsoII interaction with **60met-mC** in the presence of the cofactor analogue lacked such an increase in anisotropy (Figure 6A). Due to these findings, the anisotropy increase at time points >4–5 s in Figure 9A most likely corresponded to formation of the catalytically competent complex leading to methylation of the cytosine residue of **60met-mC**.

In the case of substrate **60met**, the initial increase in anisotropy was followed by its decrease lasting up to 20–30 s (Figure 9B). This anisotropy decrease coincided with the TAMRA fluorescence intensity increase and a subsequent decrease (Figure 8B) corresponding to the enzyme–substrate complex isomerisation and catalytically competent complex formation that precedes the methylation of one cytosine residue. At the time points after 20–30 s, TAMRA anisotropy showed an increase, reaching a plateau (Figure 9B), which coincided with the rise of fluorescence intensity in Figure 8B. Thus, the final increase in anisotropy in Figure 9B most likely corresponded to formation of the catalytically competent complex leading to methylation of the ‘second’ cytosine residue in **60met**.

After reaching the plateau phases, TAMRA anisotropy values did not return to their initial values in the kinetic series for both **60met-mC** and **60met** duplexes (Figure 9). This result indicated that we did not register the step of DNA product dissociation from its complex with the enzyme in the TAMRA anisotropy traces of **60met-mC** or **60met** during their interaction with M.SsoII in the presence of AdoMet. These data and the data on changes in TAMRA fluorescence intensity revealed that M.SsoII remained in the stable complex with the DNA product of the enzymatic reaction (monomethylated as well as dimethylated).

The anisotropy traces of **60met-mC** during its interaction with M.SsoII in the presence of AdoMet (Figure 9A) were fitted to a kinetic scheme containing four steps (Scheme 4). A step of initial enzyme–substrate complex formation was followed by two steps of isomerisation of the complex and a step of formation of the catalytically competent complex leading to the cytosine residue methylation as well as in the case of the TAMRA fluorescence traces (Figure 8A).

**Scheme 6:**
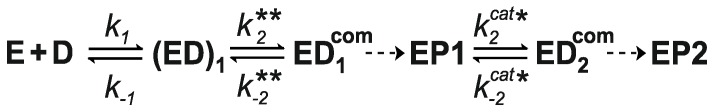
The kinetic scheme describing TAMRA anisotropy traces of substrate **60met** upon its interaction with M.SsoII in the presence of AdoMet. E is an M.SsoII molecule, D is a DNA substrate, (ED)_1_ is an enzyme–substrate complex, ${\mathrm{ED}}_{1}^{\mathrm{com}}$ and ${\mathrm{ED}}_{2}^{\mathrm{com}}$ are catalytically competent complexes, while EP1 and EP2 are the complexes of M.SsoII with the methylation reaction products: mono- and dimethylated duplexes, respectively.

At the same time, fitting the anisotropy traces of **60met** upon its interaction with M.SsoII in the presence of AdoMet (Figure 9B) yielded a kinetic scheme containing three steps (Scheme 6). The first stage represented formation of the initial enzyme–substrate complex; the second one corresponded to isomerisation of this complex and methylation of one cytosine residue, whereas the third one represented formation of the catalytically competent complex leading to methylation of the other cytosine residue. One can see that the TAMRA anisotropy traces presented in Figure 9B did not allow us to divide the steps of isomerisation of the initial enzyme–substrate complex and formation of the catalytically competent complex leading to methylation of the ‘first’ cytosine residue. Thus, kinetic parameters *k*_2_** and *k_−_*_2_** are not absolute but rather effective ones describing the joint process of isomerisation and methylation of the ‘first’ cytosine residue. Although TAMRA has been shown to be rotationally coupled to the DNA motion [69], TAMRA anisotropy traces of **60met** (Figure 9B) clearly showed that some conformational transitions in the enzyme–substrate complex were not accompanied by TAMRA anisotropy changes. Measuring the changes in TAMRA fluorescence intensity allowed us to register an additional step (which was invisible in the anisotropy traces) in the process of M.SsoII interaction with **60met** in the presence of AdoMet. The fluorescence intensity increase corresponding to this additional step is probably due to alterations in the TAMRA environment induced by the rearrangement in the enzyme globule. This rearrangement likely leads to formation of the enzyme conformation that shields TAMRA from the solution, because it is known that a decrease in TAMRA-surrounding polarity significantly increases the fluorescence intensity of a TAMRA–DNA adduct, while the fluorescence anisotropy does not change meaningfully [70].

Fitting the anisotropy traces provided the rate constant values of the M.SsoII interaction with **60met-mC** or **60met** in the presence of AdoMet (Table 4).

Taking into account the strong noise observed in the kinetic curves in the initial time slot (Figure 8 and Figure 9), we can say that the values of the corresponding rate constants for formation of the initial M.SsoII complex (*k*_1_, *k*_−1_) with **60met-mC** or **60met** in the presence of AdoMet closely approximate each other (Table 4). Thus, the number of unmethylated Cyt bases within the methylation site barely influences the initial enzyme–substrate binding in the presence of AdoMet. All kinetic parameters are discussed below in detail (see the Section 4).

### 3.8. Kinetics of Flipping out of the Target Base

Once bound to the target DNA, a MTase starts enzymatic catalysis. The first step is flipping out of the target base from the DNA double helix. It takes place even in the cases when the further catalytic steps are impossible (for example, in the presence of AdoHcy instead of AdoMet) [43,55,71,72]. To study this flipping out, analogues of DNA substrates are traditionally used where the target cytosine is replaced by 2-aPu. Such a replacement does not impair recognition of the methylation site by the MTase because the enzyme does not form specific contacts with the heterocyclic base of the target residue. In the absence of the enzyme, 2-aPu is stacked inside the DNA double helix even though it is paired with a guanine residue. Addition of the MTase leads to flipping out of 2-aPu thus increasing 2-aPu fluorescence intensity [72].

**Figure 10:**
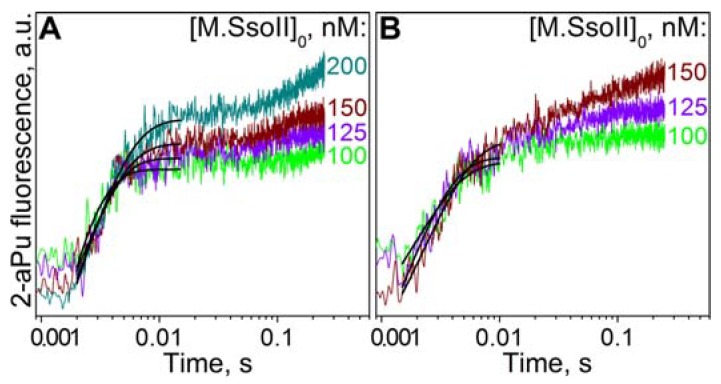
2-Aminopurine fluorescence traces of substrate **60met-mC-aPu** during its interaction with M.SsoII in the presence of AdoMet (**A**) or in the absence of a cofactor (**B**). Smooth curves are the result of the fitting procedure. Concentrations of the substrate are 100 nM. Concentrations of M.SsoII are indicated next to each curve.

We aimed to determine the rate of the target Cyt’s flipping out during M.SsoII interaction with substrates **60met-aPu** or **60met-mC-aPu** (Figure 1) in the presence of AdoMet or AdoHcy or in the absence of any of them. Figure 10 shows the representative kinetic traces for changes in 2-aPu fluorescence intensity. An increase in the fluorescence intensity takes place from time points 1.5–2 ms up to time points 10–15 ms and barely depends on the initial enzyme concentration. The kinetic traces obtained during M.SsoII interaction with monomethylated substrate **60met-mC-aPu** clearly showed that this increase did not begin from the initial detection moment. Thus, this increase most likely does not reflect formation of an initial enzyme–substrate complex but corresponds to the conformational rearrangement in this complex resulting in the flipping out of 2-aPu from the DNA double helix. Certain kinetic curves show the slow 2-aPu fluorescence intensity increase at time points beyond 10–15 ms. Such a slow increase reflects further isomerisation of the enzyme–substrate complex.

The kinetic traces presented in Figure 10 do not depict the 2-aPu fluorescence intensity changes corresponding to formation of the initial enzyme–substrate complex. Moreover, the time slot of 2-aPu’s flipping out overlaps with the time slot of the formation of the initial enzyme complex with TAMRA-labelled substrates. Besides, only the beginning of the process was observed in certain 2-aPu fluorescence traces at the time points beyond 10–15 ms. Due to these findings, there was no opportunity to perform a reliable global non-linear least-squares fitting of the kinetic traces presented in Figure 10 by means of the DynaFit software. Therefore, time slots from 1.5 or 2 ms up to 10 or 15 ms of each kinetic curve were fitted separately by one exponent in the OriginPro software. In each series of kinetic curves, a weighted average was calculated for the observed kinetic constant of the target base’s flipping out ($\bar{k}$*_fl_*) (Table 5).

## 4. Discussion

In the last decade, an increasing number of proteins has been found to be multifunctional. The most evident type of multifunctionality is based on several structural domains united in one polypeptide chain, each of them performing its own function [73]. In bacteria, bifunctional proteins composed of two domains are considered an efficient and economical way to synchronise various developmental and cell biological processes [73]. One of such proteins is M.SsoII, which regulates the functioning of the SsoII RM system.

On the one hand, M.SsoII is a transcription factor; on the other hand, it is an enzyme catalysing DNA methylation. A DNA duplex with the regulatory site is a strong inhibitor of M.SsoII methylation activity. In the present study, we proposed detailed kinetic mechanisms of M.SsoII interactions with DNA containing the methylation or regulatory site. Besides, we tried to understand the intricacies of this protein’s interactions with two different recognition sites whose concentrations in the cell differ significantly (see below).

### 4.1. Methyltransferase SsoII as an Enzyme that Modifies DNA

M.SsoII is a classical (cytosine-C5)-DNA methyltransferase. The only cysteine residue of this enzyme, Cys142, plays the key role in the catalysis of methyl group transfer onto the C5 atom of a cytosine residue [30]. The mechanism of methyl group transfer catalysed by C5-MTases has been known since 1987 [74]. At the same time, how C5-MTases recognise specific nucleotide sequences in DNA and select certain dC for methylation is still poorly studied. Here, we analysed the kinetics of M.SsoII binding to the methylation site in DNA and to a methyl group donor (AdoMet).

#### 4.1.1. The Order of the DNA Substrate and AdoMet Binding

Any DNA MTase has two substrates, DNA (unmethylated or monomethylated) and a methyl group donor (AdoMet). As to the paradigmatic enzyme M.HhaI, different binding mechanisms have been proposed, e.g., an ordered Bi Bi mechanism where DNA binds before AdoMet does [74,75] along with a random binding [76]. There is an assumption that DNA is required for AdoMet binding in the productive orientation [57,77]. Meanwhile, M.HhaI was shown to form a stable complex with the cofactor in the absence of DNA, and this complex was found to be catalytically competent [55,76].

Our results show that M.SsoII can effectively bind AdoMet in the absence of DNA (Figure 2). Vice versa, binding of M.SsoII to the DNA containing the methylation site results in fast flipping out of the target base, regardless of the cofactor presence (Figure 10). Thus, M.SsoII can bind each substrate independently of the other one, as M.HhaI can. The binding of M.SsoII to its target DNA is two orders of magnitude faster than AdoMet’s binding to M.SsoII (Table 1 and Table 4). This large difference allows us to hypothesise that M.SsoII should initially bind AdoMet and remain bound to it in the cell in order to perform efficient methylation when it finds the target DNA.

Although the total AdoMet concentration in *E. coli* is reported to be in the 300–500 µM range [78], the concentration of free AdoMet can be significantly lower due to a great number of AdoMet-binding proteins, which belong to 15 superfamilies [79]. Nevertheless, M.SsoII in the cell is likely to be in an AdoMet-bound form (*K*_d_ of 0.57 μM). The *K*_d_ values for the M.HhaI complex with AdoMet are one order of magnitude higher (the range from 4.4 to 11.5 µM in different reports) [55,56,57,80]. Thus, a 10-fold lower amount of M.SsoII in comparison with M.HhaI is necessary for effective cofactor binding.

#### 4.1.2. Kinetics of M.SsoII Interaction with DNA Substrates Containing the Unmethylated and Monomethylated CCNGG Site in the Presence of AdoMet or AdoHcy

According to our results, it appears that the rate of formation of the initial M.SsoII–DNA substrate complex does not depend on the nature of the cofactor (AdoMet or AdoHcy) and the number of unmethylated Cyt bases within the methylation site. Indeed, taking into account the strong noise observed in the kinetic curves in the initial time slot, one can state that the values of the corresponding rate constants for formation of the initial M.SsoII–**60met** complex in the presence of AdoMet and AdoHcy closely approximate each other and the values of the corresponding constants obtained in the case of **60met-mC** (Table 4). Besides, the values of the corresponding rate constants for formation of the initial M.SsoII–**60met-mC** complex in the presence of cofactor AdoMet and its analogue AdoHcy are identical within a margin of error (Table 4).

We can theorise that the second equilibrium in Scheme 3 and Scheme 4 corresponds to the isomerisation step leading to bending of the DNA backbone by ~31° (see above). This assumption is made because the kinetic parameters of this equilibrium are of the same order of magnitude as the parameters of the only isomerisation step during M.SsoII interaction with the regulatory site; this step most likely corresponded to the DNA bending (see *k*_2_, *k*_−2_ in Table 3 and Table 4).

The second equilibrium in Scheme 3 and Scheme 4 is shifted towards the post-isomerisation complex (ED)_2_ between M.SsoII and **60met-mC** in the presence of AdoMet, but towards the pre-isomerisation complex (ED)_1_ in the presence of AdoHcy (see *k*_2_, *k*_−2_ in Table 4). This difference reveals that the presence of cofactor AdoMet accelerates the induced fit leading to the DNA bending. Besides, the AdoHcy replacement by AdoMet in the reaction solution leads to an-order-of-magnitude increase in the stability of the complex (ED)_2_ (calculated as *k*_2_/*k*_−2_). At the same time, the values of the corresponding rate constants of the second isomerisation step in Scheme 3 and Scheme 4 obtained in the presence of AdoMet and AdoHcy differ negligibly (see *k*_3_, *k_−_*_3_ in Table 4), pointing to the lack of a significant influence of the cofactor nature on this step.

Although M.SsoII binding with the methylation site in the presence of cofactor analogue AdoHcy is not followed by the chemical reaction, there is an additional third isomerisation step that is absent in the case of AdoMet presence (Scheme 3). This third isomerisation step proceeds slowly because the corresponding forward and reverse rate constants (*k*_4_ and *k_−_*_4_) are rather low (Table 4). The beginning of the additional step observed in the presence of AdoHcy concurs with the beginning of the methylation step observed in the presence of AdoMet (Figure 6A, Figure 7A and Figure 8A). These data allow us to suppose that M.SsoII ‘is trying’ to carry out the methylation reaction, slowly changing its conformation even under the conditions preventing substrate methylation.

In the presence of cofactor AdoMet, the forward rate constant of formation of the catalytically competent complex leading to the ‘first’ Cyt methylation in substrate **60met** ($k_{1}^{cat}$) is sixfold higher than the forward rate constant of formation of the catalytically competent complex leading to the Cyt methylation in monomethylated substrate **60met-mC** ($k_{2}^{cat}$). The reverse rate constants for these processes barely differ (Table 4). The difference in rate constants $k_{1}^{cat}$ and $k_{2}^{cat}$ reveals that we are not able to differentiate between the formation of the catalytically competent complex and the methylation reaction of the cytosine residue. The formation of the catalytically competent complex limits the rate of this step; therefore, this step was fitted to a reversible equilibrium even though the methyl transfer reaction itself is irreversible.

Meanwhile, the forward and reverse rate constants ($k_{2}^{cat}$, $k_{-2}^{cat}$) of catalytically competent complex formation leading to Cyt methylation in **60met-mC** (which is also a ‘second’ Cyt) are on average fivefold and twofold higher than the corresponding rate constants of the process preceding to ‘second’ Cyt methylation in **60met** ($k_{2}^{cat}$*, $k_{-2}^{cat}$*; Table 4). These data can be explained as follows: formation of the ‘second’ catalytically competent complex occurs after methylation of one Cyt and before methylation of the other Cyt, therefore contributing to the obtained rate constants ($k_{2}^{cat}$*, $k_{-2}^{cat}$*) of the ‘second’ Cyt methylation in **60met**. The rate constants $k_{2}^{cat}$* and $k_{-2}^{cat}$* describe the joint process of formation of the ‘second’ catalytically competent complex and methylation of the ‘second’ cytosine residue.

Thus, we demonstrated in vitro the possibility of sequential methylation of two cytosine residues in opposite strands of one methylation site. At the same time, we cannot rule out the in vivo scenario where the enzyme molecule that has just methylated the ‘first’ cytosine is displaced from the binding site on DNA by another cellular protein.

The kinetic traces obtained during the interaction of M.SsoII with the methylation site (**60met-mC-aPu** or **60met-aPu**) detecting changes in 2-aPu fluorescence intensity revealed the conformational rearrangement in the initial enzyme–substrate complex leading to the flipping out of 2-aPu from the DNA double helix. The time slot of 2-aPu’s flipping out overlaps with the time slot of formation of the initial enzyme complex with TAMRA-labelled substrates; the values of the observed kinetic constants for the target base flipping out ($\bar{k}$*_fl_*) are rather high (Table 5). Because the 2-aPu residue in DNA duplexes used in our study substitutes the target Cyt, we believe that regardless of the nature of a cofactor (AdoMet or AdoHcy), the target Cyt flips out very rapidly immediately after M.SsoII’s binding to the substrate.

Our kinetic data allow us to theorise that the target Cyt’s flipping out occurs prior to the bending of the DNA backbone in the complex of M.SsoII with the methylation site. This notion is in agreement with the observation that DNA bending is not a prerequisite for base flipping [81]. Existence of an initial enzyme–DNA complex where the DNA base is flipped into the enzyme’s active site in the absence of significant DNA bending has been demonstrated for uracil-DNA glycosylase (UNG), which engulfed a thymine into its active site pocket [82].

On the basis of the whole set of the data obtained, we propose the kinetic mechanism of processing of the DNA substrate containing the methylation site by M.SsoII in the presence of AdoMet or AdoHcy. Irrespective of the nature of the cofactor (AdoMet or AdoHcy) present in the reaction mixture, M.SsoII rapidly generates the initial enzyme–substrate complex (ED)_1_, in which the target Cyt immediately flips out, and the generated complex undergoes the conformational transition into (ED)_2_ leading to bending of the DNA backbone (Scheme 3, Scheme 4 and Scheme 5). In the presence of AdoHcy, the (ED)_2_ complex undergoes two conformational transitions into (ED)_3_ and (ED)_4_ ‘trying’ to carry out the methylation reaction (Scheme 3). In the presence of AdoMet, the (ED)_2_ complex undergoes one conformational transition into (ED)_3_, which corresponds to the induced fit (Scheme 4). Next, formation of the catalytically competent complex ${\mathrm{ED}}_{1}^{\mathrm{com}}$ leading to methylation of the ‘first’ cytosine residue occurs resulting in formation of the M.SsoII complex with the reaction product EP1 (Scheme 5 and Scheme 6). We suppose that this first methylation step is followed by the formation of the ‘second’ catalytically competent complex ${\mathrm{ED}}_{2}^{\mathrm{com}}$ with subsequent methylation of the ‘second’ cytosine residue (EP2) (Scheme 4, Scheme 5 and Scheme 6). After that, the process of the dissociation of the enzyme–product complex takes place. The enzyme–product complex is probably rather stable, thus the terminal dissociation step likely proceeds slowly.

### 4.2. Methyltransferase SsoII as a Transcription Factor

M.SsoII forms a specific and stable complex with the promoter region of the genes of the SsoII RM system. This protein binds to the 15-mer inverted repeat inside the promoter region and forms specific contacts with the trinucleotides 5′-GGA-3′ and 5′-TGT-3′, which are located within the inverted repeat symmetrically relative to the central A/T pair [33]. Such symmetrical interaction with both halves of the recognition site is typical for many regulatory proteins that function as dimers with their operator sequences and utilise α-helices introduced into the DNA major groove for site recognition. In this study, we propose a kinetic scheme of M.SsoII interaction with the regulatory site and describe a detailed mechanism of gene transcription regulation in SsoII-like RM systems.

#### 4.2.1. Kinetics of M.SsoII Interaction with DNA Duplexes Containing the Regulatory Site

The kinetic mechanism of M.SsoII interaction with the regulatory site includes at least two reversible steps. The first step represents the initial protein–DNA complex formation, while the second step is isomerisation of this complex leading to bending of the DNA backbone (Scheme 2). The DNA undergoes a bend of ~45° in complex with the M.SsoII monomer and ~90° in complex with the M.SsoII dimer (Table 2). The following rule is observed for the M.SsoII monomer binding to each duplex containing the regulatory site used in this study (**60reg-1**, **60reg-2** or **60reg-4**): the forward and reverse rate constants of the initial protein–DNA binding (*k*_1_ and *k*_−1_) have the lowest values in the presence of cofactor analogue AdoHcy, intermediate values in the presence of cofactor AdoMet and the highest values without either (Table 3). Thus, these results reveal that the presence of a cofactor or its analogue in the solution delays the forward and reverse reaction of formation of the initial protein–DNA complex.

#### 4.2.2. Do (A/T)_4_ Tracts Flanking the Regulatory Site Influence the M.SsoII Binding

The intergenic region of the SsoII RM system contains many poly(A/T) tracts, which are typical for gene promoters. Among them, two (A/T)_4_ tracts flank the regulatory site (Figure 1, duplex **60reg-1**). (A/T)_4_ tracts are known to induce DNA bending by 9° [83]. We examined their role in the M.SsoII interaction with the regulatory site. M.SsoII shows the most effective binding to duplex **60reg-1** where the (A/T)_4_ tracts are located just as in the natural sequence of the SsoII RM system. Inverting the (A/T)_4_ tracts (as in **60reg-2**) increases the amount of complex **C1** and decreases that of complex **C2**. Deletion of the (A/T)_4_ tracts (as in **60reg-3**) only slightly decreases the amount of complex **C1** in the range of M.SsoII excess over DNA from 2- to 20-fold (Figure S5). Thus, M.SsoII can sense slight differences in DNA conformation which are caused by the presence or absence of (A/T)_4_ tracts in the regions flanking the regulatory site.

Nevertheless, the bending angles induced by M.SsoII in the **162reg-1** and **162reg-2** duplexes are the same within the margin of error (Table 2). Most likely, the angles formed by the (A/T)_4_ tracts lie in different planes in comparison with the angles induced by M.SsoII. Besides, the corresponding kinetic parameters of M.SsoII’s initial binding to duplexes **60reg-1** and **60reg-2** and the subsequent induced fit barely depend on the orientation of the (A/T)_4_ tracts (Table 3). Given such a small difference in the complex formation and the similar bending angles, we can conclude that the (A/T)_4_ tracts flanking the regulatory site play no important role in the functioning of M.SsoII in the cell.

#### 4.2.3. The Role of DNA Bending by M.SsoII in Transcriptional Regulation of the Genes in SsoII-Like RM Systems

**Figure 11:**
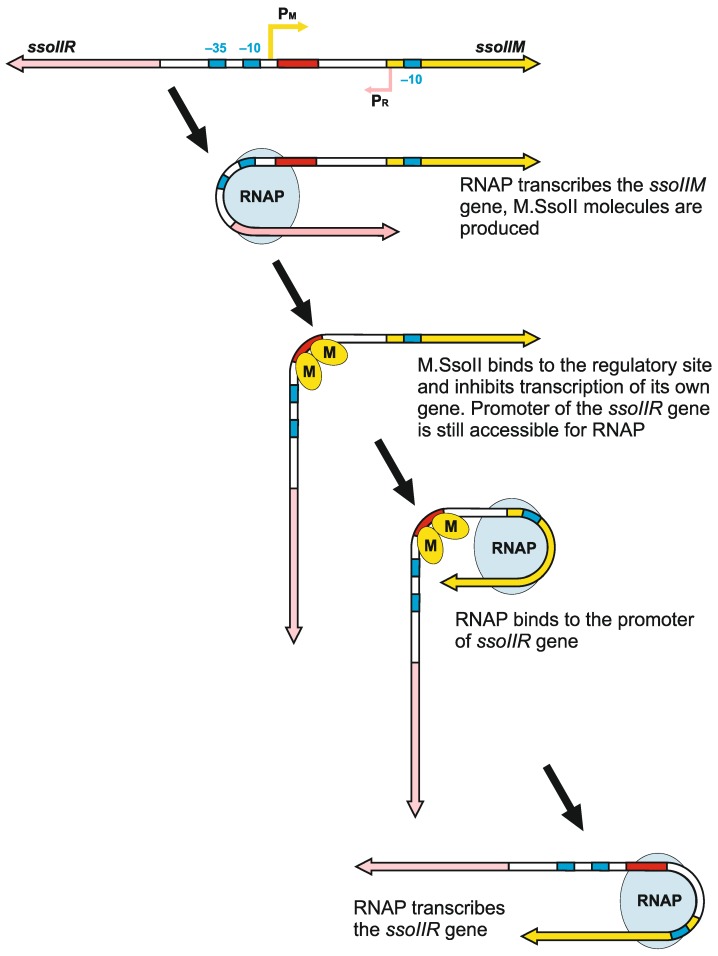
The role of DNA bending in transcription regulation in the restriction–modification system SsoII. M.SsoII is shown in yellow and marked with a letter M. RNA polymerase (RNAP) is light blue. The gene *ssoIIM* (yellow) encodes M.SsoII; its transcription initiation point is marked as P_M_. Gene *ssoIIR* (pink) encodes R.SsoII; its transcription start site is marked as P_R_. The −10 and −35 promoter elements are in blue. The regulatory site is in red. As demonstrated in the present work, the M.SsoII dimer binds to the regulatory site and induces DNA bending by 90°. RNAP is well known to induce overall DNA bending of ~180° [21].

The general scheme of transcription regulation in the SsoII-like RM systems has been described elsewhere [84,85]. The details of this model on the basis of our data are presented in Figure 11 (Supplementary Information). Penetration of a SsoII RM system into a cell is followed by active transcription of the *ssoIIM* gene by RNA polymerase (RNAP) because the promoter of this gene is reported to be stronger than the *ssoIIR* gene promoter [84]. With time, a certain amount of M.SsoII molecules is synthesised that protects the host cell genomic DNA and the plasmid carrying the RM system from R.SsoII hydrolysis. (This time was at least 90 min in the case of RM system Esp1396I [86]). Then, two M.SsoII molecules bind to the regulatory site, bend it by ~90° (Table 2) and block the access of RNAP to the *ssoIIM* gene promoter. The interaction of the M.SsoII C-terminal domain with the DNA flanking the regulatory site described elsewhere, [35], apparently provides the additional stability to the DNA−protein complex (Figure 11).

Because the regulatory site is located closer to the MTase promoter than to the REase promoter, the steric competition between M.SsoII and RNAP for the binding site was suggested [85]. Our results on bending of the regulatory site by M.SsoII and the DNA−protein complex isomerisation support this notion. RNAP is known to induce an overall bend of ~180° in DNA [21]. According to the model described previously [21], the DNA region from −11 to +20 is placed in the active site channel of RNAP and is therefore inaccessible to any other protein. Yet the regulatory site is located from +7 to +21 relative to the transcription start site of the *ssoIIM* gene. Thus, M.SsoII and RNAP have overlapping binding sites; they both induce strong bending in DNA, and their simultaneous binding to adjacent sites is sterically impossible (Figure 11).

In contrast, the distance between the *ssoIIR* promoter and the regulatory site is slightly longer. The site is located between +34 and +48 relative to the transcription start site of the *ssoIIR* gene, and this space is enough to allow both M.SsoII and RNAP to bind to DNA (Figure 11). When transcription starts from the *ssoIIR* promoter, RNAP displaces M.SsoII from the regulatory site during elongation; the collision with M.SsoII does not impair transcription in this case. Thus, the M.SsoII binding to the regulatory site indirectly leads to the activation of the REase gene promoter due to prevention of RNAP binding to the MTase gene promoter [31,84]. Probably the bend in the regulatory region that we observed brings M.SsoII and σ-subunit of RNAP together thus stimulating the REase gene transcription.

### 4.3. How Does Methyltransferase SsoII Combine Its Two Mutually Exclusive Functions?

DNA methylation is the main M.SsoII function. Yet the DNA duplex with the regulatory site is a strong inhibitor of the M.SsoII methylation activity. Moreover, binding of M.SsoII to the regulatory site in the promoter region of the SsoII RM system prevents production of new M.SsoII molecules. How does M.SsoII combine its two functions that seem to be mutually exclusive?

According to our estimation (see Section 1), a cell should containabout 2 × 10^4^ methylation sites, while amount of the regulatory sites should be equal to amount of plasmids encoding the SsoII RM system. Importantly, the SsoII RM system is dangerous for the host cell when the cell receives the RM system for the first time. In case M.SsoII does not modify the methylation sites fast enough, R.SsoII can cleave them and multiple double-strand breaks in DNA could be lethal for the cell. As soon as all the methylation sites are fully methylated by M.SsoII, the SsoII RM system becomes quite safe for the host cell, since R.SsoII does not cleave methylation sites which are methylated in one or both DNA strands. Thus, the bottleneck for spreading of the SsoII RM system is the moment when a cell receives the plasmid encoding the RM system for the first time. Most likely, the host cell receives only one such plasmid during conjugation. Thus, we can assume that the cell contains only one regulatory site in the critical moment, which means having the methylation sites in a 20,000-fold excess. Such an excess is large enough to ensure M.SsoII’s binding to the methylation sites in the first place.

Moreover, our exploration of the kinetics of M.SsoII interaction with DNA ligands indicates that the catalytically competent M.SsoII complex with the methylation site forms more efficiently than the M.SsoII complex with the regulatory site in the presence of cofactor AdoMet. We clearly demonstrated that the replacement of AdoHcy with AdoMet—in the case of M.SsoII interaction with the substrate containing the methylation site—shifts the equilibrium towards the complex (ED)_2_ which is characterised by the DNA bending; and increases stability of this complex 10-fold (calculated as *k*_2_/*k*_−2_; see *k*_2_, *k*_−2_ in Table 4). In contrast, such a replacement does not significantly influence the forward and reverse rate constants of the isomerisation (*k*_2_ and *k*_−2_) for the initial M.SsoII complexes with the duplexes containing the regulatory site (Table 3).

On the other hand, our kinetic data indicate that the duplex containing the regulatory site is more preferable for M.SsoII than the substrate containing the methylation site at the step of formation of the initial protein–DNA complex. This situation ensures efficient formation of the M.SsoII complex with the regulatory site. Indeed, the formation rates (*k*_1_) of the initial M.SsoII complexes [(ED)_1_] with duplexes containing the regulatory site are slightly higher (on average 1.4-fold in the presence of AdoHcy as well as AdoMet) than in the case of the substrates containing the methylation site (Table 3 and Table 4). Furthermore, the stability values of (ED)_1_ complexes (calculated as *k*_1_/*k*_−1_) are higher for the duplexes containing the regulatory site than for the substrates containing the methylation site (on average threefold in the presence of AdoHcy and twofold in the presence of AdoMet). Besides, the formation rate and stability of complex (ED)_1_ are higher in the case of the regulatory site in the absence of AdoMet and AdoHcy than even in the case of the methylation site in the presence of a cofactor or its analogue.

## 5. Conclusions

M.SsoII is an effective natural switch that regulates functioning of the SsoII RM system. Here we report an analysis of the mechanisms of M.SsoII interactions with the DNA containing regulatory or methylation sites. Our kinetic study reveals that M.SsoII can bind DNA containing the methylation site and the methyl group donor AdoMet independently of each other. In the presence of AdoMet or its metabolic product AdoHcy, M.SsoII rapidly generates an initial complex with the methylation site (containing two unmethylated cytosine residues). The latter undergoes several conformational transitions leading to the bending of the DNA backbone by ~31° and formation of the catalytically competent enzyme–substrate complexes in the presence of AdoMet.

The kinetic mechanism of M.SsoII interaction with the regulatory site includes the initial protein–DNA complex formation and single isomerisation caused a DNA backbone bend (of ~45° in complex with the M.SsoII monomer and ~90° in complex with the M.SsoII dimer).

Kinetic data indicate that M.SsoII prefers the regulatory, not the methylation site, at the step of formation of the initial enzyme–DNA complex. On the other hand, the induced fit in the presence of cofactor AdoMet is accelerated in the M.SsoII complex with the methylation site, but not the regulatory site. A large excess of the methylation sites over the regulatory sites in the cell also promotes the binding of M.SsoII to the methylation sites in the first place. Taken together, these factors provide efficient DNA methylation by M.SsoII despite the strong inhibitory action of the regulatory site.

## Figures and Tables

**Table 1:** Rate constants for M.SsoII interactions with AdoMet.

Ligand	*k*_1_, μM*^−^*^1^·s*^−^*^1^	*k*_−1_, s*^−^*^1^	*k*_2_, s*^−^*^1^	*k*_−2_, s*^−^*^1^
AdoMet	6.9 ± 0.3	93 ± 4	7.0 ± 0.3	7.9 ± 0.3

The indicated errors represent standard deviations of theoretically fitted plots from the stopped-flow curves. Actual error values also involve experimental errors, not exceeding 20%.

**Table 2:** Angles of DNA bending induced by M.SsoII binding to its target sites in 162-bp DNA duplexes.

DNA–Protein Complex	DNA Bending Angle, Degrees	Number of Experiments	*R*^2^_adjusted_ **
**162reg-1 + M.SsoII**	46 ± 6 *	5	0.739–0.974
**162reg-1 + 2M.SsoII**	89 ± 5	6	0.946–0.996
**162reg-2 + M.SsoII**	43 ± 6	6	0.714–0.909
**162reg-2 + 2M.SsoII**	88 ± 4	6	0.966–0.998
**162met + M.SsoII**	31 ± 9	5	0.595–0.948

* The indicated errors represent standard deviations. ** *R*^2^_adjusted_ is the adjusted coefficient of determination.

**Table 3:** Rate constants for M.SsoII interactions with DNA duplexes containing the regulatory site.

DNA Duplex	Cofactor	*k*_1_, μM*^−^*^1^·s*^−^*^1^	*k*_−1_, s*^−^*^1^	*k*_2_, s*^−^*^1^	*k*_−2_, s*^−^*^1^
**60reg-1**	AdoMet	390 ± 8	93 ± 1	1.29 ± 0.04	11.0 ± 0.4
	AdoHcy	352 ± 7	71 ± 1	2.30 ± 0.04	12.8 ± 0.3
	—	470 ± 10	127 ± 1	1.14 ± 0.05	10.0 ± 0.5
**60reg-2**	AdoMet	390 ± 12	88 ± 1	1.13 ± 0.02	9.6 ± 0.2
	AdoHcy	350 ± 11	52 ± 1	1.18 ± 0.02	7.6 ± 0.2
	—	430 ± 11	109 ± 1	1.06 ± 0.02	11.1 ± 0.2
**60reg-4**	AdoMet	386 ± 7	154 ± 1	0.68 ± 0.02	6.5 ± 0.3
	AdoHcy	342 ± 5	110 ± 1	1.15 ± 0.02	7.5 ± 0.2
	—	468 ± 9	195 ± 1	0.69 ± 0.03	5.4 ± 0.4

The indicated errors represent standard deviations of theoretically fitted plots from the stopped-flow curves. Actual error values also involve experimental errors, not exceeding 20%.

**Table 4:** Rate constants for M.SsoII interactions with DNA duplexes containing the methylation site.

	AdoHcy				AdoMet			
	60met-mC		60met		60met-mC		60met	
	Figure 6A, TAMRA Anisotropy Analysis	Figure 7A, TAMRA Fluorescence Analysis	Figure 6B, TAMRA Anisotropy Analysis	Figure 7B, TAMRA Fluorescence Analysis	Figure 9A, TAMRA Anisotropy Analysis	Figure 8A, TAMRA Fluorescence Analysis	Figure 9B, TAMRA Anisotropy Analysis	Figure 8B, TAMRA Fluorescence Analysis
*k*_1_, μM*^−^*^1^·s*^−^*^1^	226 ± 3	226 ^a^	181 ± 3	324 ± 4	223 ± 4	230 ± 4	341 ± 7	338 ± 5
*k*_−1_, s*^−^*^1^	158 ± 1	158 ^a^	143 ± 1	159 ± 1	183 ± 2	139 ± 1	254 ± 3	162 ± 1
*k*_2_ *, s*^−^*^1^	0.22 ± 0.01		0.40 ± 0.02					0.18 ± 0.04
*k*_−2_ *, s*^−^*^1^	0.50 ± 0.02		1.5 ± 0.1					0.25 ± 0.16
*k*_2_ **, s*^−^*^1^							0.025 ± 0.003	
*k*_−2_ **,s*^−^*^1^							0.094 ± 0.007	
*k*_2_, s*^−^*^1^		3.0 ± 0.1			7.0 ± 0.2	7.6 ± 0.1		
*k*_−2_, s*^−^*^1^		13.0 ± 0.1			3.7 ± 0.2	3.1 ± 0.1		
*k*_3_, s*^−^*^1^		0.48 ± 0.004			0.46 ± 0.06	0.64 ± 0.05		
*k*_−3_, s*^−^*^1^		0.21 ± 0.002			0.31 ± 0.03	0.19 ± 0.02		
*k*_4_, s*^−^*^1^		0.0015 ± 0.0001						
*k*_−4_, s*^−^*^1^		0.0078 ± 0.0009						
$k_{1}^{cat}$, s*^−^*^1^								0.36 ± 0.02
$k_{-1}^{cat}$, s*^−^*^1^								0.053 ± 0.006
$k_{2}^{cat}$, s*^−^*^1^					0.068 ± 0.001	0.057 ± 0.001		
$k_{-2}^{cat}$, s*^−^*^1^					0.045 ± 0.001	0.030 ± 0.001		
$k_{2}^{cat}$*, s*^−^*^1^							0.013 ± 0.001	0.018 ± 0.001
$k_{-2}^{cat}$*, s*^−^*^1^							0.017 ± 0.001	0.021 ± 0.001

**^a^** The values of kinetic constants *k*_1_ and *k*_−1_ were assumed to be equal to the corresponding rate constants obtained during fitting of the anisotropy kinetic traces. The asterisks * and ** label effective kinetic parameters describing the joint processes but not the individual kinetic steps. The indicated errors represent standard deviations of theoretically fitted plots from the stopped-flow curves. Actual error values also involve experimental errors, not exceeding 20%.

**Table 5:** Observed rate constants for the target base’s flipping out upon M.SsoII interactions with DNA duplexes containing the methylation site.

	60met-mC-aPu			60met-aPu		
	AdoMet	AdoHcy	-	AdoMet	AdoHcy	-
$\bar{k}$***_fl_***, s^−1^	483 ± 10	472 ± 8	494 ± 14	416 ± 12	640 ±33	408 ± 29

The indicated errors represent standard deviations of weighted averages. Actual error values also involve experimental errors, not exceeding 20%.

## Supplementary Materials

The supplementary materials are available online.

## Abbreviations

2-aPu	2-Aminopurine
AdoHcy	*S*-Adenosyl-l-homocysteine
AdoMet	*S*-Adenosyl-l-methionine
Cyt	Cytosine residue
EMSA	Electrophoretic mobility shift assay
MTase	DNA methyltransferase
M.HhaI	DNA methyltransferase HhaI
M.SsoII	DNA methyltransferase SsoII
PAGE	Polyacrylamide gel electrophoresis
R.SsoII	Restriction endonuclease SsoII
REase or R.	Restriction endonuclease
RM	Restriction–modification
RNAP	RNA polymerase
