# Supplementary material for: Kinetic Basis of the Bifunctionality of SsoII DNA Methyltransferase
Source: Molecules. 2018 May 16;23(5):1192. doi: 10.3390/molecules23051192 (PMC6100179; doi:10.3390/molecules23051192)
Supplement: Supplementary file 1 [file molecules-23-01192-s001.zip › molecules-295714-SI.pdf]

The kinetic basis of bifunctionality of SsoII DNA methyltransferase

Nadezhda A. Timofeyeva, Alexandra Yu. Ryazanova, Maxim V. Norkin, Tatiana S. Oretskaya, Olga S. Fedorova, Elena A. Kubareva

SUPPLEMENTARY INFORMATION

Mutagenesis of plasmid pBend2

Because plasmid pBend2 [1] contains two instances of the R.SmaI recognition sequence (5′-CCCGGG-3′/3′-GGGCCC-5′), and this sequence includes the M.SsoII methylation site (5′-CCNGG-3′/3′-GGNCC-5′), the R.SmaI recognition sequence has to be removed from the plasmid. The removal was performed by several rounds of site-directed mutagenesis (Figure S1). Each R.SmaI site was changed into 5′-CACGTG-3′/3′-GTGCAC-5′. The mutagenesis was performed according to the scheme of Ho *et al.* [2], which is based on internal and external primers. The internal primers (F1 and R1) are complementary to each other and contain the desired mutation. The external primers (F2 and R2) flank the whole template sequence. Two PCRs (one with the primers F2 and R1, the other one with the primers F1 and R2) produce two DNA fragments which overlap in the region of the mutated site. These two DNA fragments are further hybridised and used as a template for PCR with the external primers (F2 and R2) in order to obtain the desired long PCR product with mutation. In our case, the procedure was complicated because both R.SmaI sites were located in an identical sequence context. Therefore, we repeated the mutagenesis according to the scheme of Ho *et al.* [2] twice (the products shown in Figure S1A were used as a template for PCRs shown in Figure S1B).

According to the scheme of Ho *et al.* [2], we used the initial plasmid pBend2 as a template in two PCRs (Figure S1A). The following primers were used: 5′-CCAGCTGCACGTGAGGCCTTC-3′ (F1), 5′-GAAGGCCTCACGTGCAGCTGG-3′ (R1), 5′-CGTATCACGAGGCCCTTTCG-3′ (F2), and 5′-AACGCGCGAGGCAGATC-3′ (R2). PCR-1 (with the primers F2 and R1) yielded a 228-bp DNA fragment with the desirable mutation in one of the two R.SmaI sites (the ‘right’ site) and a 106-bp side product. The 228-bp fragment was easily separated from the shorter side product by gel electrophoresis. PCR-2 (with the primers F1 and R2) yielded a 350-bp DNA fragment with the desirable mutation in one of the two R.SmaI sites (the ‘left’ site) and a 230-bp side product. The 350-bp fragment and 228-bp fragment were extracted from the gel after the electrophoretic separation. The 228-bp and 350-bp fragments have an overlapping region of 142 bp and each fragment carries a mutation in one of the two R.SmaI sites. Two DNA polymerases were tried for each PCR, Taq DNA Polymerase and Phusion High-Fidelity DNA Polymerase (Thermo Scientific, USA). Taq DNA polymerase provided a higher yield for the 228-bp fragment, while Phusion polymerase was more effective for the 350-bp fragment synthesis. The reaction mixture for Taq polymerase (20 µl) contained 1× Taq buffer, Taq DNA polymerase (1 U), dNTP (150 µM), $MgCl_2$ (1.5 mM), forward and reverse primers (0.5 µM each) and DNA template (10 ng). PCR mixture for Phusion polymerase (20 µl) contained 1× TF buffer, Phusion DNA polymerase (0.4 U), dNTP (150 µM), DMSO (3%), forward and reverse primers (0.5 µM each), and DNA template (10 ng). The PCR conditions were 95°C for 2 min (1 cycle) followed by 30 cycles of 95°C, 60°C, and 72°C for 30 s each. The fragments were purified by electrophoresis in a 2% agarose gel followed by extraction with the PCR Clean-Up and Gel Extraction Kit (Macherey-Nagel, Germany).

The 228-bp and 350-bp fragments were mixed and reannealed in a thermocycler (6 cycles of 95°C and 72°C for 30 s each). Then the external primers (F2 and R2) were added and new PCR was performed (PCR-3 in Figure S1A). The PCR conditions were 95°C for 2 min (1 cycle) followed by 22 cycles of 95°C, 60°C, and 72°C for 30 s each. It should be noted that the final mixture contained four variants of the 436-bp fragment where each DNA strand carried one desirable mutation (the sequence 5′-CCCGGG-3′ changed to 5′-CACGTG-3′).

Then the mutagenesis scheme of Ho *et al.* [2] was repeated once again (Figure S1B). The mixture of

436-bp fragments was used as a template in PCR-4 (with the primers F2 and R1) to produce a mixture of new 228-bp DNA fragments (four variants which differed in the position of the desired mutation, see Figure S1B). Analogously, the mixture of 436-bp fragments was used as a template in PCR-5 (with the primers F1 and R2) to produce a mixture of new 350-bp DNA fragments (four variants which differed in the position of the desired mutation, see Figure S1B).

The newly obtained 228-bp and 350-bp fragments were mixed and reannealed in a thermocycler. Then the external primers (F2 and R2) were added, and a new PCR was performed (PCR-6 in Figure S1B). The final mixture contained four variants of the 436-bp fragment where each DNA strand carried one or two desirable mutations (the sequence 5′-CCCGGG-3′ changed to 5′-CACGTG-3′ in one or both DNA strands).

The resulting mixture of 436-bp fragments was cleaved by R.EcoRI and R.HindIII simultaneously to prepare a 240-bp fragment. This fragment was purified by electrophoresis in a 2% agarose gel. The initial pBend2 plasmid was also digested with R.EcoRI and R.HindIII, and the products were separated by electrophoresis in a 1% agarose gel. The linearised plasmid was ligated with the 240-bp fragment. The ligation mixture was used to transform chemically competent *E. coli* DH5α cells. Colonies were grown on agar plates with 100 μg/ml ampicillin, and five clones were further analysed by PCR with the external primers (F2 and R2). The length of the PCR products (436 bp) confirmed the presence of the insert in each plasmid. These PCR products were treated with R.SmaI in order to hydrolyse all non-mutated R.SmaI sites. The reaction products were separated by electrophoresis in a 2% agarose gel. Out of five clones checked, three contained the desired mutations at both R.SmaI sites and therefore were resistant to R.SmaI, while 2 clones were cleaved by R.SmaI. A plasmid from one of the R.SmaI-resistant clones was amplified and analysed by sequencing to confirm the presence of the desired mutations. The modified plasmid was named pBend2-Mod.

A

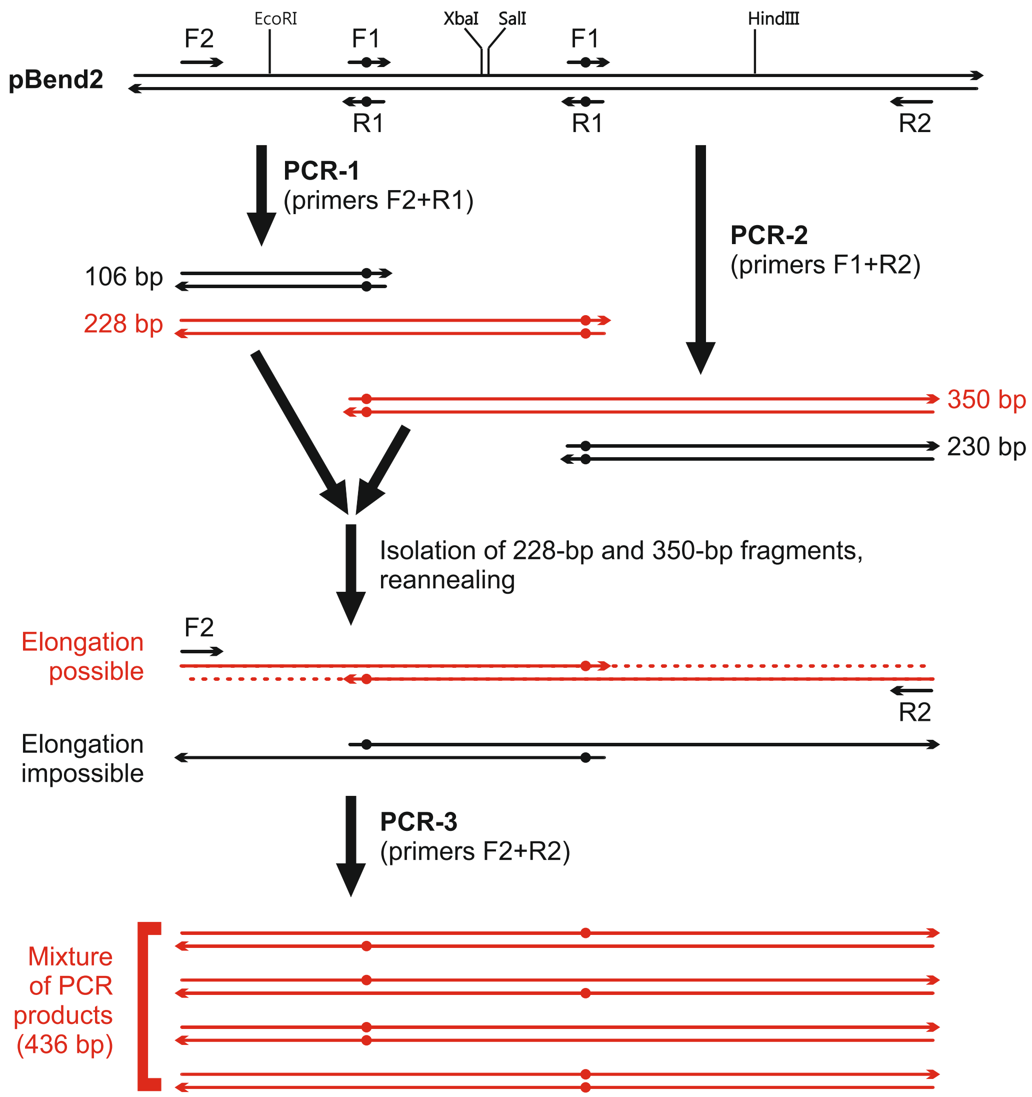

EcoRI
XbaI
SalI
HindIII
F2
F1
F1
pBend2
R1
R1
R2
PCR-1
(primers F2+R1)
PCR-2
(primers F1+R2)
106 bp
228 bp
350 bp
230 bp
Isolation of 228-bp and 350-bp fragments, reannealing
Elongation possible
F2
R2
Elongation impossible
PCR-3
(primers F2+R2)
Mixture of PCR products (436 bp)

B

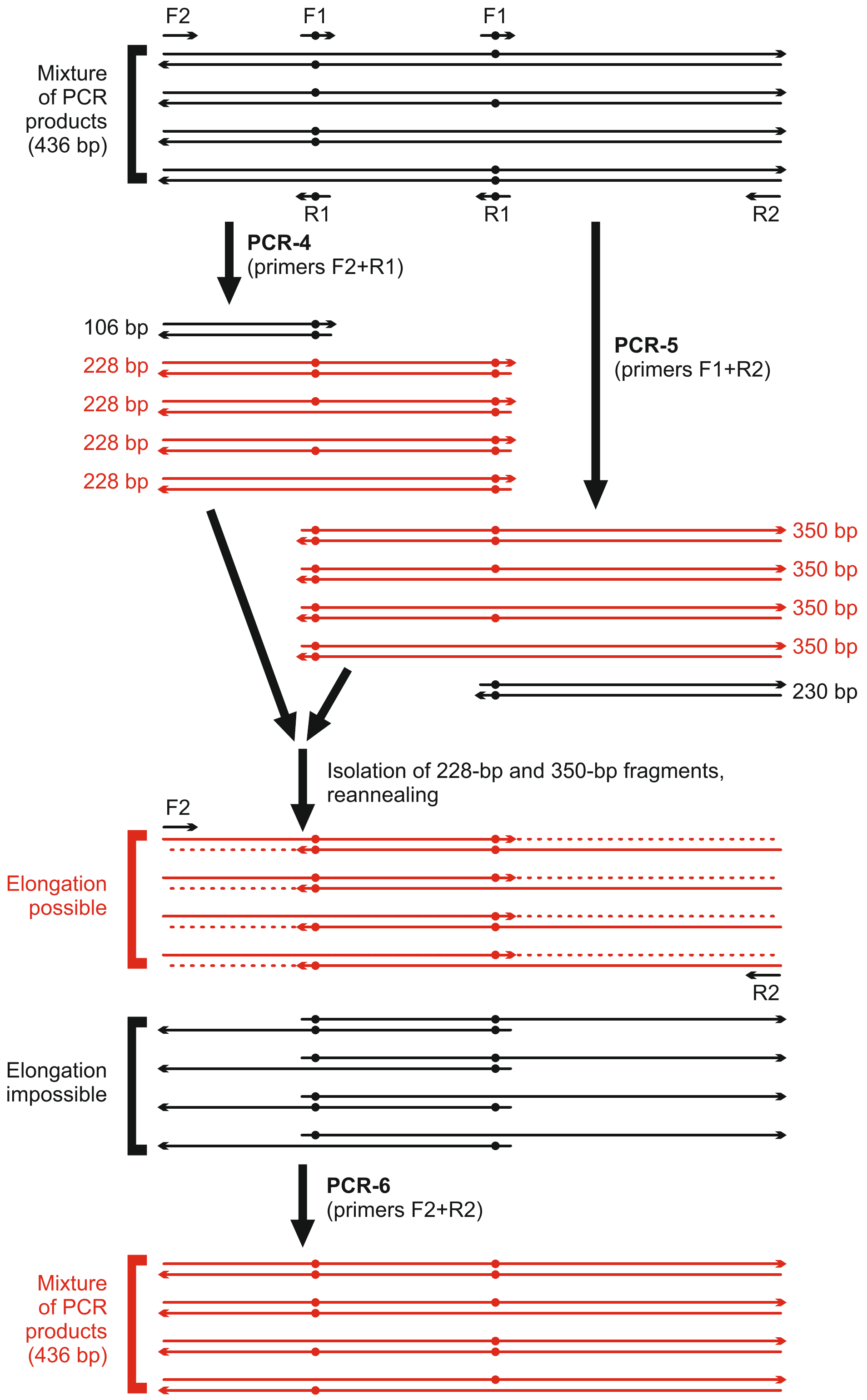

Figure S1. The mutagenesis scheme (see detailed description in the text) including the following steps: from PCR-1 to PCR-3 (**A**) and from PCR-4 to PCR-6 (**B**). Short arrows represent primers; dots stand for the mutations in the R.SmaI sites. The reaction products that are used as starting material at the next step of the overall process are highlighted in red. The positions of the R.EcoRI, R.XbaI, R.SalI, and R.HindIII cleavage sites in the pBend2 plasmid are indicated with lowercase letters (panel **A**).

Production of DNA fragments of the same length with the target site at different positions

The pBend2-Mod plasmid was hydrolysed with R.XbaI and R.SalI in order to produce sticky ends (Figure S2A). A synthetic DNA duplex with the same sticky ends was ligated into the plasmid. The first variant of this duplex contained the regulatory site with the native flanking sequence [(A/T)$_4$ tracts], the second variant contained the regulatory site with the inverted (A/T)$_4$ tracts [i.e. (T/A)$_4$ tracts], while the third variant contained the methylation site.

```
5'-CTAGATTGGAATCAAAACAGGACAAATTGTCCTAAAACCAACACTTG———3'    Reg1
3'———TAACCTTAGTTTTGTCCTGTTTAACAGGATTTTGGTTGTGAACAGCT-5'

5'-CTAGATTGGAATCTTTTCAGGACAAATTGTCCTTTTTCCAACACTTG———3'    Reg2
3'———TAACCTTAGAAAAGTCCTGTTTAACAGGAAAAAGGTTGTGAACAGCT-5'

5'-CTAGAGATAGTATGAAGCTAGAGCCAGGTTGGCAGCATTCTACTCAG———3'    Met
3'———TCTATCATACTTCGATCTCGGTCCAACCGTCGTAAGATGAGTCAGCT-5'
```

The length of each insert after ligation was 41 bp. Each ligation mixture was used to transform chemically competent *E. coli* DH5α cells. Colonies were grown on agar plates with 100 µg/ml ampicillin. The presence of the proper insert was confirmed by PCR with the external primers (F2 and R2) and by sequencing.

The plasmid with the insert was used as a template in PCR with the external primers (F2 and R2) in order to obtain a 477-bp fragment (436 + 41 = 477). This fragment was hydrolysed with a set of REases to obtain a set of linear 162-bp fragments which contained one M.SsoII recognition site (regulatory or methylation) at different positions (Figure S2A). The following REases were used: R.MluI, R.BglII, R.NheI, R.SpeI, R.XhoI, R.EcoRV, R.StuI, R.SspI, R.BamHI, R.SalI in a mixture with R.EcoRI and R.XbaI in the mixture with R.HindIII. Each 477-bp fragment was cleaved into three fragments, their lengths are indicated in Figure S2A. The target 162-bp fragments were purified by electrophoresis in a 2% agarose gel followed by isolation with the PCR Clean-Up and Gel Extraction Kit (Macherey-Nagel, Germany).

Because R.SspI and R.BamHI hydrolysed the 477-bp fragment into the fragments of similar lengths (114, 162 and 201 bp for R.SspI; 139, 162 and 176 bp for R.BamHI) which were poorly separable by electrophoresis, a more complicated DNA cleavage procedure was used in these cases (Figure S2B). The 477-bp fragment was digested with R.EcoRI and R.HindIII, the product was purified by gel extraction, cleaved with either R.SspI or R.BamHI, and purified once again.

A

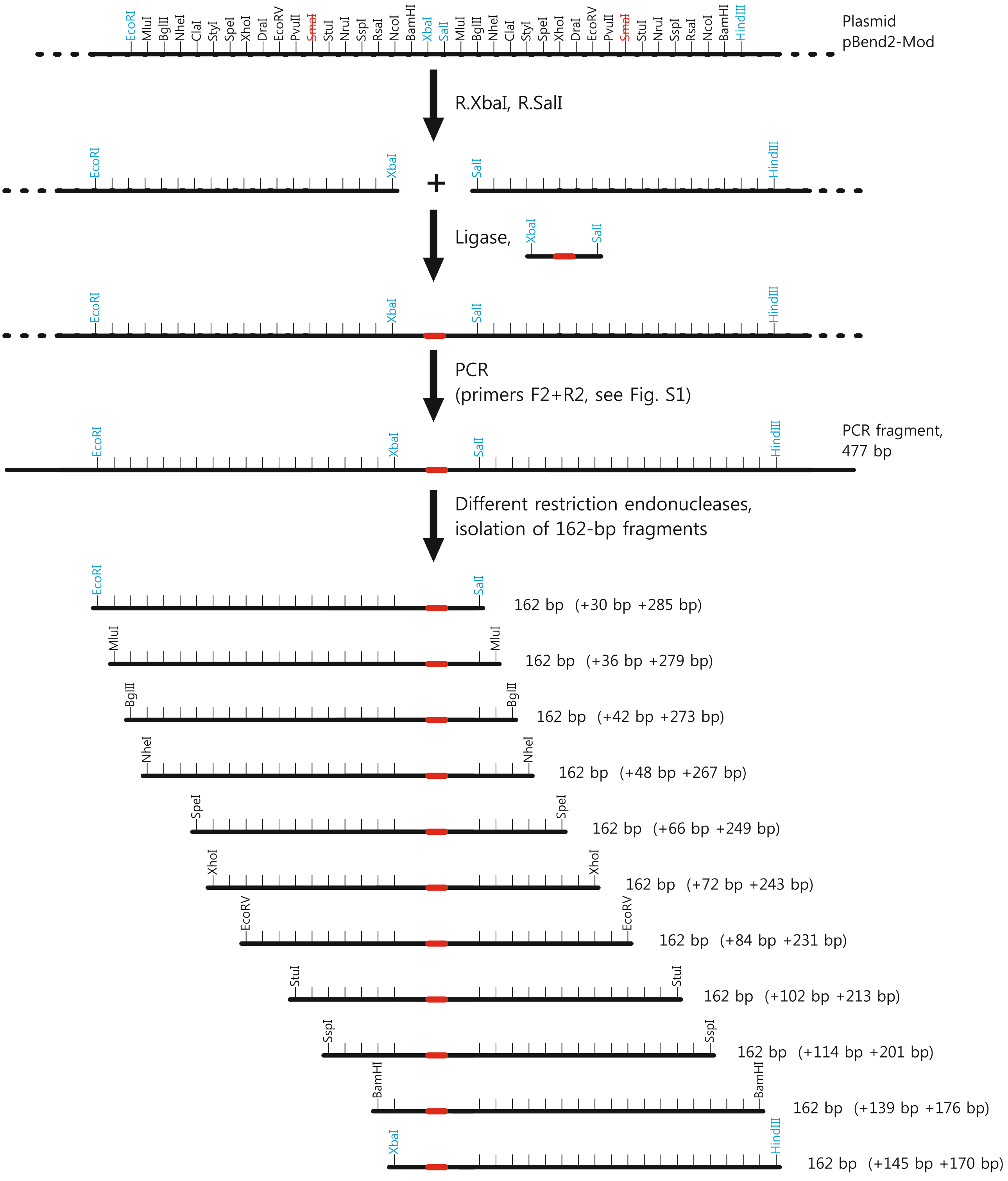
Plasmid
pBend2-Mod
EcoRI
MluI
BglII
NheI
ClaI
StyI
SpeI
XhoI
DraI
EcoRV
PvuII
SmaI
StuI
NruI
SspI
RsaI
NcoI
BamHI
XbaI
SalI
HindIII
R.XbaI, R.SalI
+
Ligase,
PCR
(primers F2+R2, see Fig. S1)
PCR fragment,
477 bp
Different restriction endonucleases,
isolation of 162-bp fragments
162 bp (+30 bp +285 bp)
162 bp (+36 bp +279 bp)
162 bp (+42 bp +273 bp)
162 bp (+48 bp +267 bp)
162 bp (+66 bp +249 bp)
162 bp (+72 bp +243 bp)
162 bp (+84 bp +231 bp)
162 bp (+102 bp +213 bp)
162 bp (+114 bp +201 bp)
162 bp (+139 bp +176 bp)
162 bp (+145 bp +170 bp)

B

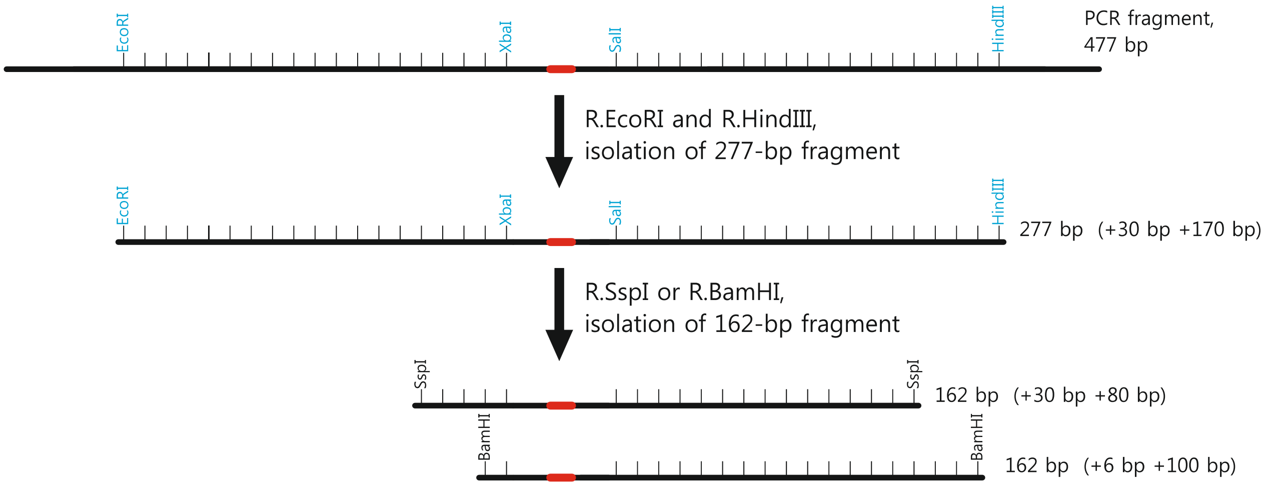

Figure S2. Preparation of a set of 162-bp fragments with the target site at different positions (see detailed description in the text). (**A**) The pBend2-Mod plasmid was cleaved with R.XbaI and R.SalI in order to obtain sticky ends. A synthetic DNA duplex with the same sticky ends was ligated into the plasmid. PCR from the resulting plasmid (with the primers F2 and R2) yielded the 477-bp product. This PCR product was cleaved with different REases. Each cleavage yielded three products, the target product of 162 bp and two side products of different lengths (indicated in brackets). The 162-bp fragment can be easily separated from the side products by gel electrophoresis, except for the case of cleavage by R.SspI and R.BamHI. (**B**) A more complicated cleavage scheme was used in the case of R.SspI and R.BamHI. At first, the 477-bp fragment was cleaved with R.EcoRI and R.HindIII, the product was isolated from the gel after electrophoretic separation, cleaved by either R.SspI or R.BamHI, and purified once again.

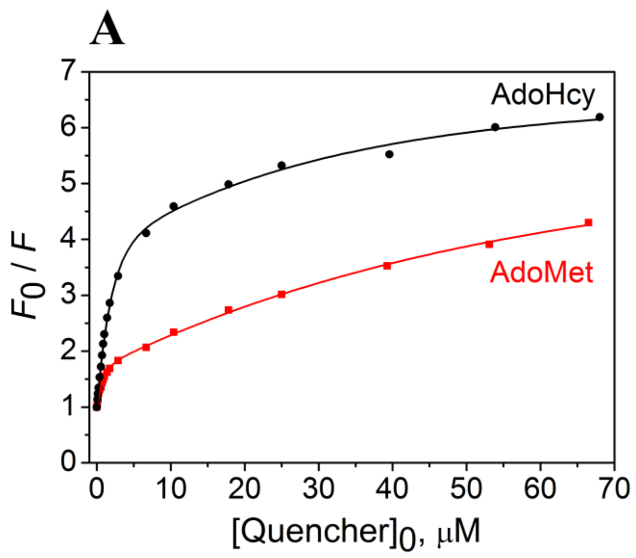

Figure S3. Quenching of Trp fluorescence in M.SsoII molecule upon its binding to AdoMet or AdoHcy. (**A**) Dependence of the relative Trp fluorescence on the quencher concentration. F_0 and F are the fluorescence intensity values in the absence and presence of the quencher, respectively. The non-linearity of the curves indicates heterogeneity of the Trp population and requires the use of the modified Stern–Volmer equation [5] (**B**) Linearisation of the Trp fluorescence quenching according to the modified Stern–Volmer equation: $F_0/(F_0 - F) = 1/(f_a K_{SV}[Q]) + 1/f_a$, where F_0 and F are the fluorescence intensity values in the absence or presence of the quencher, f_a is the fraction of fluorophores accessible for the quencher in the total fluorescence, [Q] is the quencher concentration and K_{SV} is the quenching constant. In our case, $K_{SV} = K_a = 1/K_d$ for the M.SsoII complex with AdoMet or AdoHcy. The value of the adjusted coefficient of determination ($R^2_{adj.}$) is shown near each line.

Heterogeneity of the Trp population:

Molecule of M.SsoII contains three Trp residues: one is located in the cavity of active center and two are buried inside of protein globule. Therefore, we had in mind that Trp residues have different environment and Trp residue of the active center is more accessible for fluorescence quenchers AdoMet or AdoHcy.

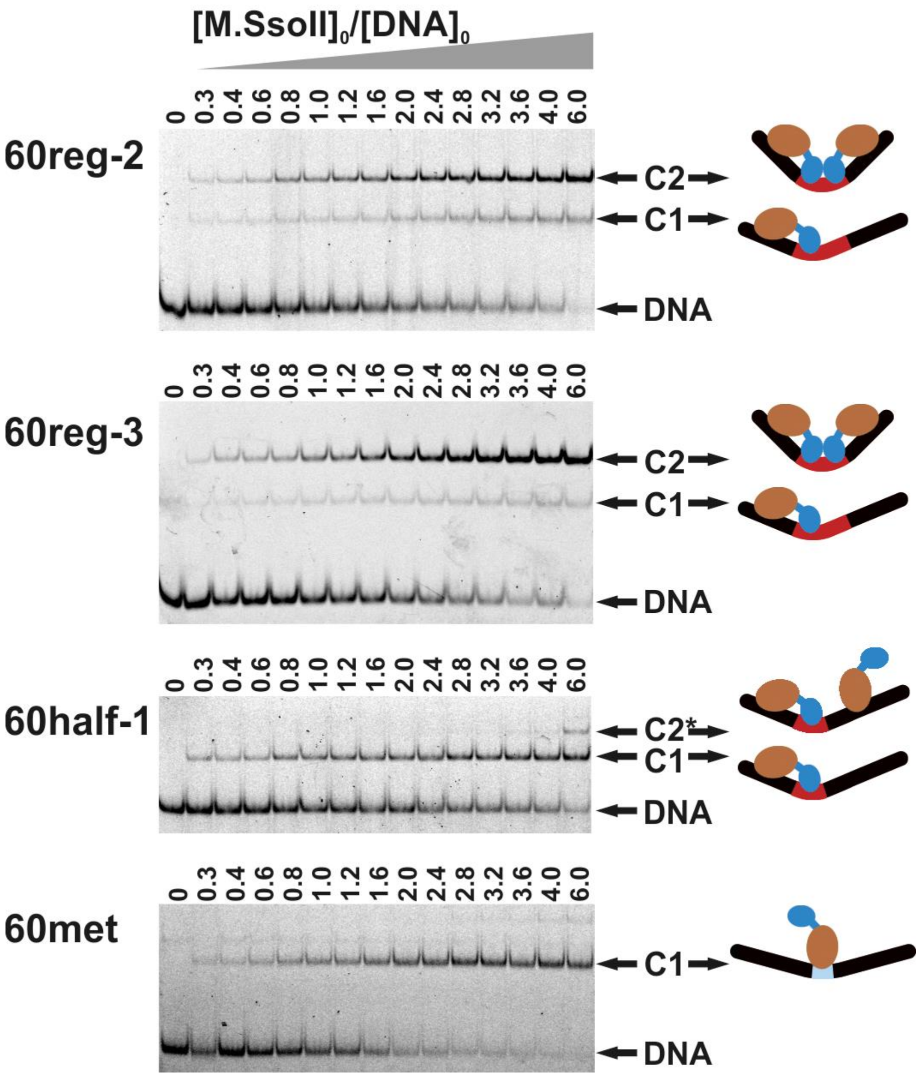

Figure S4. Complex formation between M.SsoII and various TAMRA-labelled 60-bp DNA duplexes (indicated on the left) analysed by EMSA in a non-denaturing 7% polyacrylamide gel. Results for the duplexes **60reg-2**, **60reg-3**, **60half-1**, and **60met** are shown. The ratio of M.SsoII initial concentration to DNA initial concentration is shown above the lanes. DNA concentration is 2.5 nM, M.SsoII concentration varies from 0.75 to 15 nM. Complex **C1** consists of one M.SsoII subunit and one DNA duplex. Complex **C2** consists of two M.SsoII subunits bound to the regulatory site and one DNA duplex. Complex **C2*** presumably contains one DNA duplex, one M.SsoII subunit bound specifically to the regulatory half-site and the other M.SsoII molecule bound to the flanking sequence in a non-specific manner. Structures of the complexes are presented schematically on the right taking into account the angles of DNA bending determined in this work. DNA is black, the regulatory site is red, and the methylation site is light blue. The N-terminal domain of M.SsoII is blue; the C-terminal domain is brown.

Figure S5 represents quantitative results of gel electrophoresis in polyacrylamide gel (see Section 3.3 and Figure 3 on pages 11-12). TAMRA fluorescence intensity was registered. The error bars represent standard deviation for each experimental point obtained from 3 identical independent experiments. The experimental points are connected by splines in order to improve visual clarity of the data. These splines do not correspond to any general equation.

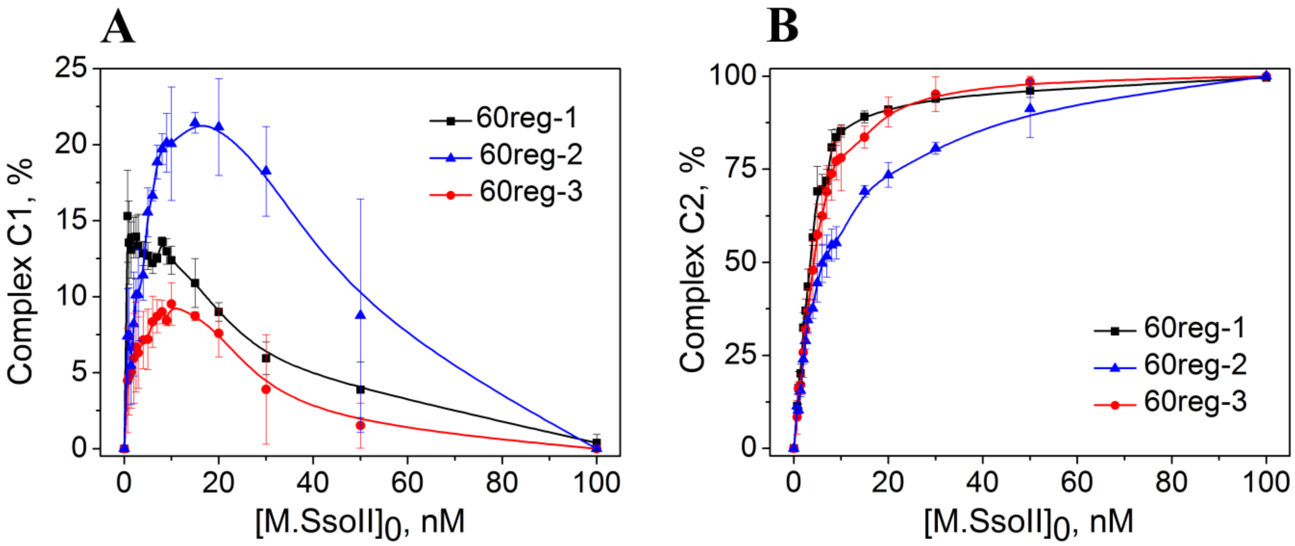

Figure S5. Quantitative differences in complex formation between **60reg-1**, **60reg-2** and **60reg-3**. TAMRA-labelled DNA (2.5 nM) is titrated with increasing M.SsoII concentrations (0.75–100 nM). (**A)** Formation of complex **C1** consisting of one M.SsoII molecule and one DNA duplex. **(B)** Formation of complex **C2** consisting of two M.SsoII subunits and one DNA duplex. The bars represent values of standard deviation (SD).

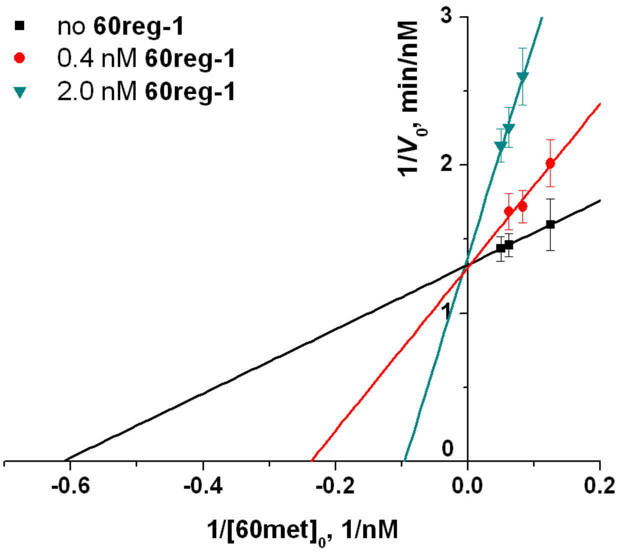

Figure S6. Inhibition of M.SsoII enzymatic activity by DNA with the regulatory site. Reaction mixtures contained 4 nM M.SsoII, 10 μM AdoMet, and from 8 to 20 nM TAMRA-labelled **60met**. The apparent K_M values obtained from the Lineweaver–Burk double-reciprocal plot are 1.6 nM in the absence of **60reg-1**, 4.2 nM in the presence of 0.4 nM **60reg-1** (ratio **60reg-1**/M.SsoII = 0.1), and 10 nM in the presence of 2.0 nM **60reg-1** (ratio **60reg-1**/M.SsoII = 0.5). The lines intersect on the $1/V_0$ axis, thus indicating a competitive mechanism of inhibition. Indeed, the long duplex **60reg-1** binds to both domains of the M.SsoII molecule and therefore occupies the catalytic site [3] preventing it from binding to **60met**. In contrast, a 31-bp duplex with the regulatory site was found to be a non-competitive inhibitor of the SsoII-like MTase Ecl18kI [4]. Presumably, the 31-bp duplex was not long enough to occupy the catalytic site and therefore it had another mechanism of inhibition and was a significantly less potent inhibitor.

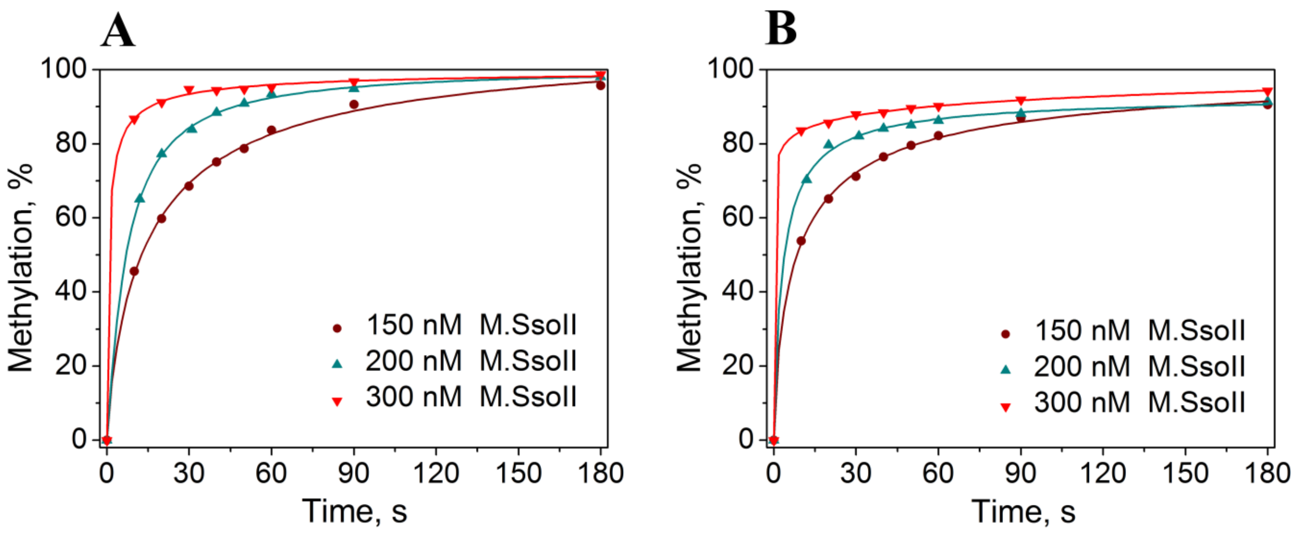

Figure S7. Methylation of duplexes **60met** (**A**) and **60met-mC** (**B**) by M.SsoII under the conditions identical to those in the stopped-flow experiments. The final mixture contained 100 nM DNA, 150–300 nM M.SsoII, and 100 μM AdoMet in buffer D (50 mM Tris–HCl, 100 mM NaCl, 5 mM β-mercaptoethanol, pH 7.5) at 25°C. Efficiency of DNA methylation was assessed on the basis of the DNA protection from hydrolysis by R.Bme1390I (see paragraph 'Methylation assay' in the section 'MATERIALS AND METHODS'). The components of the reaction were mixed by hand-pipetting.

REFERENCES

1. Kim, J.; Zwieb, C.; Wu, C.; Adhya, S. Bending of DNA by gene-regulatory proteins: construction and use of a DNA bending vector. *Gene* **1989**, 85, (1), 15-23.
2. Ho, S.N.; Hunt, H.D.; Horton, R.M.; Pullen, J.K.; Pease, L.R., Site-directed mutagenesis by overlap extension using the polymerase chain reaction. *Gene* **1989**, 77**,** (1), 51-59.
3. Ryazanova, A.Y.; Winkler, I.; Friedhoff, P.; Viryasov, M.B.; Oretskaya, T.S.; Kubareva, E.A. Crosslinking of (cytosine-5)-DNA methyltransferase SsoII and its complexes with specific DNA duplexes provides an insight into their structures. *Nucleosides Nucleotides Nucleic Acids* **2011**, 30, (7-8), 632-650.
4. Nikitin, D.V.; Mokrishcheva, M.L.; Solonin, A.S. Binding of DNA methyltransferase M.Ecl18kI [corrected] to operator-promoter region decreases its methylating activity. *Biochemistry (Mosc)* **2012**, 77**,** (3), 307-311.
5. Lakowicz, J.R., *Principles of fluorescence spectroscopy*. 3rd ed. *Springer,* **2006**, 277-289.
